# CUDC907, a dual phosphoinositide-3 kinase/histone deacetylase inhibitor, promotes apoptosis of NF2 Schwannoma cells

**DOI:** 10.18632/oncotarget.28254

**Published:** 2022-07-19

**Authors:** Julianne Huegel, Christine T. Dinh, Maria Martinelli, Olena Bracho, Rosa Rosario, Haley Hardin, Michael Estivill, Anthony Griswold, Sakir Gultekin, Xue-Zhong Liu, Cristina Fernandez-Valle

**Affiliations:** ^1^Burnett School of Biomedical Sciences, College of Medicine, University of Central Florida, Orlando, FL 32827, USA; ^2^Department of Otolaryngology, University of Miami Miller School of Medicine, Miami, FL 33136, USA; ^3^John P. Hussman Institute for Human Genomics, University of Miami Miller School of Medicine, Miami, FL 33136, USA; ^4^Department of Pathology, University of Miami Miller School of Medicine, Miami, FL 33136, USA; ^5^Department of Human Genetics, University of Miami Miller School of Medicine, Miami, FL 33136, USA

**Keywords:** fimepinostat, Schwann cell, vestibular schwanomma, merlin, nerve allograft

## Abstract

Neurofibromatosis Type 2 (NF2) is a rare tumor disorder caused by pathogenic variants of the merlin tumor suppressor encoded by *NF2*. Patients develop vestibular schwannomas (VS), peripheral schwannomas, meningiomas, and ependymomas. There are no approved drug therapies for NF2. Previous work identified phosphoinositide-3 kinase (PI3K) as a druggable target. Here we screened PI3K pathway inhibitors for efficacy in reducing viability of human schwannoma cells. The lead compound, CUDC907, a dual histone deacetylase (HDAC)/PI3K inhibitor, was further evaluated for its effects on isolated and nerve-grafted schwannoma model cells, and primary VS cells. CUDC907 (3 nM IG_50_) reduced human merlin deficient Schwann cell (MD-SC) viability and was 5–100 fold selective for MD over WT-SCs. CUDC907 (10 nM) promoted cell cycle arrest and caspase-3/7 activation within 24 h in human MD-SCs. Western blots confirmed a dose-dependent increase in acetylated lysine and decreases in pAKT and YAP. CUDC907 decreased tumor growth rate by 44% in a 14-day treatment regimen, modulated phospho-target levels, and decreased YAP levels. In five primary VS, CUDC907 decreased viability, induced caspase-3/7 cleavage, and reduced YAP levels. Its efficacy correlated with basal phospho-HDAC2 levels. CUDC907 has cytotoxic activity in NF2 schwannoma models and primary VS cells and is a candidate for clinical trials.

## INTRODUCTION

Neurofibromatosis type 2 (NF2) is a genetic disorder that causes growth of multiple benign tumor types throughout the central and peripheral nervous systems. NF2 patients develop bilateral vestibular schwannomas (VS) that cause hearing loss, facial weakness, imbalance, and potentially fatal brainstem compression [1, 2]. Patients can develop other peripheral and cranial schwannomas as well as multiple meningiomas and ependymomas. Standard treatment options for NF2-associated VS are microsurgical resection and radiation therapy; however, both carry the risk of facial nerve damage and deafness. Surgical resection of tumors in the brain, spinal cord, and peripheral nerves carry similar inherent risks due to their location. Radiotherapy also increases the risk for malignant transformation and development of secondary neoplasms in the radiation field [3, 4]. Cumulative effects of the tumors and their clinical interventions result in morbidity that severely reduces quality of life and shortens life expectancy for NF2 patients [5–7]. Although an anti-vascular endothelial growth factor (VEGF) monoclonal antibody, bevacizumab, demonstrated partial tumor regression in 41% and improved hearing in 20% of NF2 patients, its chronic use is limited by renal toxicity [8]. Phase II studies of the epidermal growth factor receptor (EGFR) inhibitor, lapatinib, and the mammalian target of rapamycin (mTOR) inhibitor, everolimus, have been conducted based on pre-clinical success; lapatinib showed some promising response in a limited number of NF2 patients, though these results are thought to be due primarily to anti-proliferative effects [9, 10]. Currently, there are no approved drug therapies for NF2. The need for cytotoxic treatments to provide tumor shrinkage and impactful long-term control is clear.

NF2 is caused by pathogenic variants in the *NF2* gene that encodes merlin, an actin-associated tumor suppressor [11, 12]. Merlin acts as a scaffold protein to regulate cell adhesion, motility, proliferation, and survival downstream of receptor activation [13, 14]. Among these are: receptor tyrosine kinases, integrins, and cadherins that converge on common intracellular kinases, including large tumor suppressor kinase (LATS, part of the Hippo pathway), mitogen-activated protein kinase (MAPK), and phosphoinositide-3 kinase (PI3K) / protein kinase B (AKT) / mTOR [14–18]. Efforts to identify an effective small molecule inhibitor targeting a single kinase have led to NF2 clinical trials in children and adults [9, 10]. Inhibitors of histone deacetylase (HDAC), mTOR, anaplastic lymphoma kinase (ALK), mitogen-activated protein kinase kinase (MEK), and receptor tyrosine kinases have been or are being evaluated for NF2. However, targeting a single kinase with the expectation of significant and persistent tumor control as is needed in this multiple tumor disorder is proving untenable, particularly given the complexity and extent of signaling pathways modulated by merlin.

We conducted an unbiased chemical compound screen of the Library of Pharmacologically Active Compounds (LOPAC) as a pilot high-throughput screen to identify NF2 schwannoma targets for inhibition. The screen identified PI3K as a lead vulnerable pathway [19]. This follow-up screen of nearly 200 PI3K pathway inhibitors identified CUDC907, a dual class 1 PI3K and HDAC inhibitor as a lead drug [20, 21]. Resistance to HDAC inhibitors is associated with activation of PI3K signaling and was a driving factor in development of a small molecule inhibitor with dual activities [21, 22]. CUDC907 (fimepinostat) is in clinical trials for children and young adults with central nervous system tumors (NCT02909777, NCT03893487) [23]. It has fast-track designation for adults with diffuse large B cell lymphoma with *MYC* pathogenic variants following a phase 2 trial (NCT02674750). Here, we report that CUDC907 reduced viability of multiple human schwannoma cell models and cells from five primary human VS, and slowed tumor growth in an orthotopic allograft mouse model. Moreover, CUDC907 promoted caspase-dependent death in all schwannoma models studied. Cytotoxicity is a desired endpoint as it suggests that CUDC907 could reduce tumor burden in NF2 patients as opposed to preventing tumor progression.

## RESULTS

### Unbiased high-throughput screen of 174 PI3K/AKT/mTOR/ inhibitors identified CUDC907 as a candidate therapeutic

**Figure 1:**
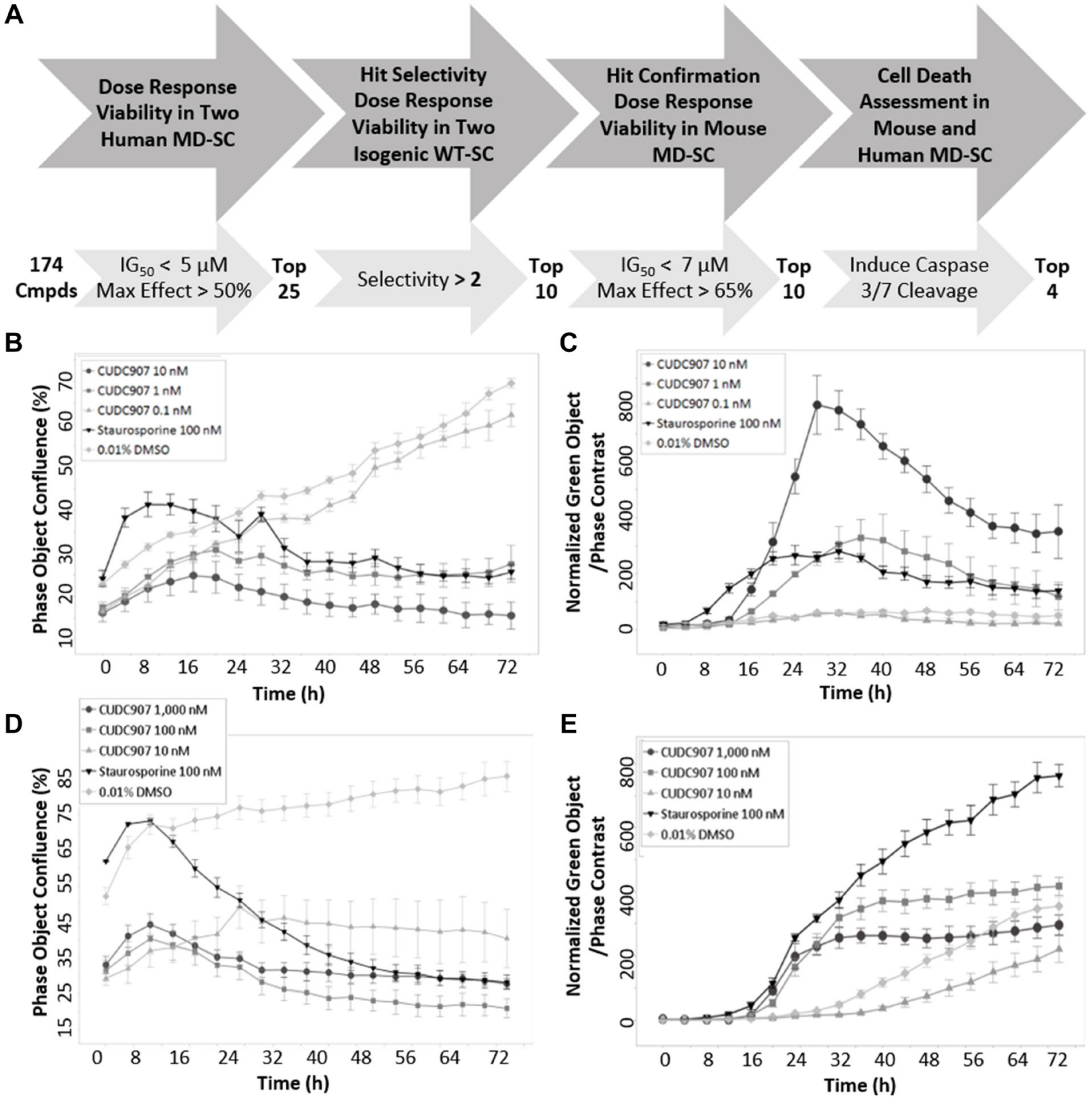
Identification criteria for CUDC907. (**A**) Summary of workflow and criteria used to screen 174 PI3K/mTOR/AKT inhibitors in human and mouse MD-SCs. Time course of (**B**) growth and (**C**) caspase cleavage in human MD-SCs. Time course of (**D**) growth and (**E**) caspase cleavage in mouse MD-SCs. CUDC907 concentrations indicated in figures. CUDC907 has a nanomolar IG_50_ and activates caspase 3/7 in human and mouse MD-SC.

Screening of 174 small molecule inhibitors of the PI3K/AKT/mTOR pathway (Supplementary Table 1) was performed to identify drugs that diminished human MD-SC viability. Selection criteria is summarized in Figure 1A. Cell viability was measured using two assays in two human MD-SC lines over a concentration range of 0.1 nM to 10 μM, with a target IG_50_ less than 5 μM and a maximum effect greater than 50%; 25 inhibitors met those criteria. Selectivity was measured in the two isogenic wild-type (WT) normal human SC lines. Ten inhibitors demonstrated 2–100 fold selectivity for MD-SCs over WT-SCs. These inhibitors also significantly diminished viability of mouse MD-SC with low IG_50_ (less than 7 μM) and high maximum effect (greater than 65%). The final screening criterion was induction of cell death measured with a live imaging caspase-3/7 cleavage assay. Only four of the ten inhibitors triggered caspase-3/7 cleavage, and only one, CUCD907, is in clinical trial (fimepinostat). The four inhibitors that met screening criteria are identified with bold text in Supplementary Table 1. Viability data for all human and mouse cell lines treated with CUDC907 is provided (Table 1). CUDC907 reduced proliferation of human MD-SCs (HS01) in a dose dependent manner (Figure 1B) that coincided with the appearance of cleaved caspase-3/7 positive cells (Figure 1C) over 72 h. These results were confirmed in mouse MD-SC (MS01, Figure 1D and 1E).

**Table 1:** Efficacy and selectivity of CUDC907 in merlin-deficient schwann cells

Cell Line	Primary Assay: CellTiter-Fluor			Secondary Assay: CyQuant		
	IG_50_ (nM)	Maximum Effect (%)	Fold Selectivity (MD/WT)	IG_50_ (nM)	Maximum Effect (%)	Fold Selectivity (MD/WT)
**HS01/HS11 (MD/WT-HSC)**	3/60	83/85	20	1/10	90/91	10
**HS05/HS13 (MD/WT-HSC)**	5/500	86/68	111	2/10	94/91	5
**MS01 (MD-MSC)**	660	80	N/A	490	80	N/A

### CUDC907 promoted cell cycle arrest and apoptosis of HS01 cells

**Figure 2:**
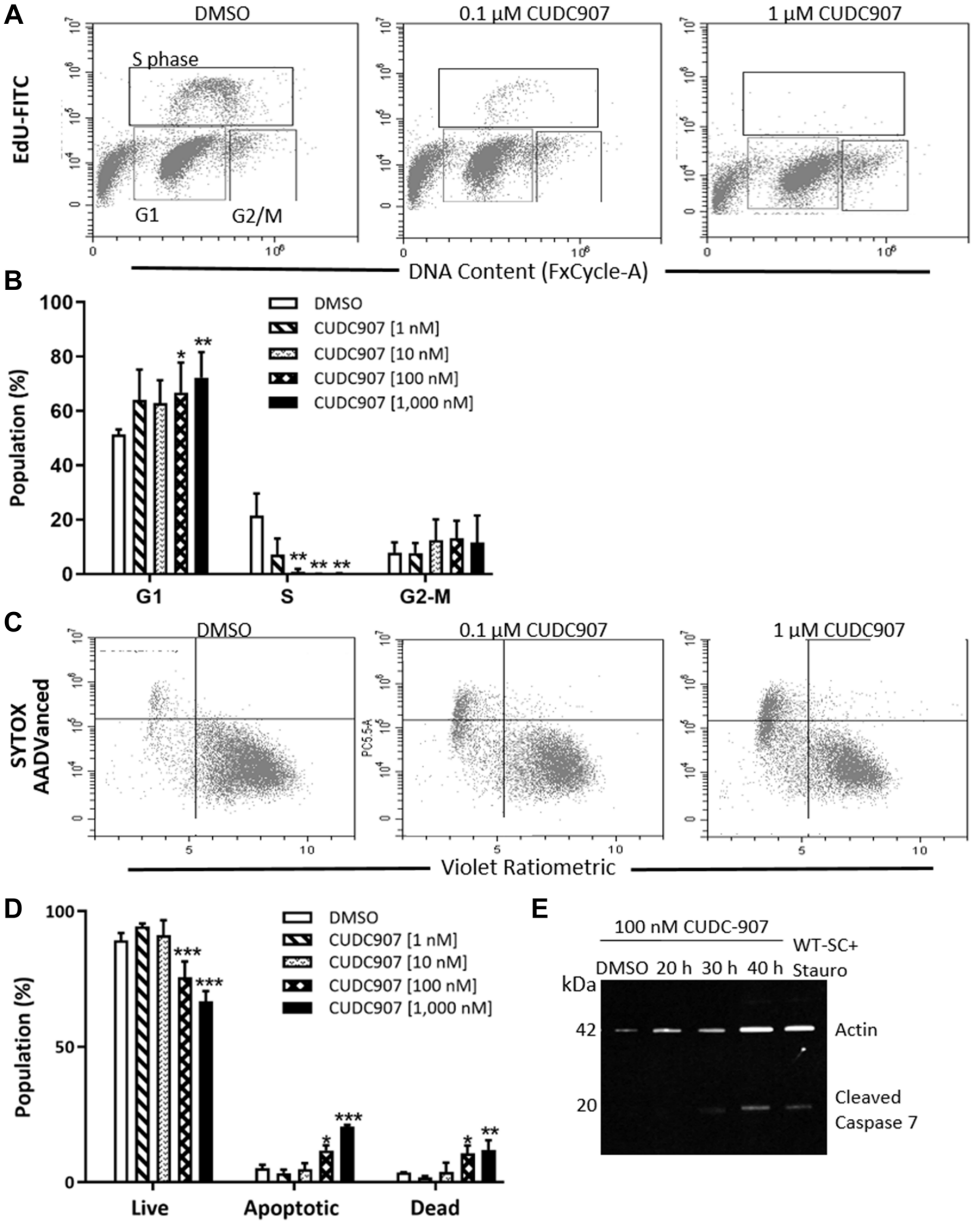
CUDC907 promotes cell cycle arrest and apoptosis of human MD-SC *in vitro*. (**A**) Representative result of cell cycle analysis of HS01 cells treated with CUDC907 for 24 h. (**B**) Quantitation of three independent experiments. (**C**) Representative plots of membrane asymmetry and (**D**) quantitation of three independent experiments of HS01 cells treated with CUDC907 for 24 h. (**E**) Representative Western blot of HS01 treated as indicated. Data in B and D are mean ± SD of three independent experiments. ^*^ *p* < 0.05, ^**^ *p* < 0.01, ^***^ *p* < 0.0001 compared to DMSO.

Cell cycle analysis of human MD-SCs (HS01) treated with 0.1 and 1 μM CUDC907 for 24 h revealed a significant increase in G1 phase cells with a concurrent reduction in S phase cells (Figure 2A and 2B). CUDC907 did not alter the percentage of cells in the G2-M phase. Membrane asymmetry measured by flow cytometry confirmed significant increases in both apoptotic and dead cell populations after 24 h of treatment with both 0.1 and 1 μM CUDC907 compared to DMSO controls (Figure 2C and 2D). Western blots revealed cleaved caspase-7, but not cleaved caspase-3, in whole cell lysates of HS01 cells exposed to 0.1 μM CUDC907 for 30 h (Figure 2E and data not shown).

### CUDC907 inhibited PI3K and HDAC activity and reduced YAP levels in HS01 cells

**Figure 3:**
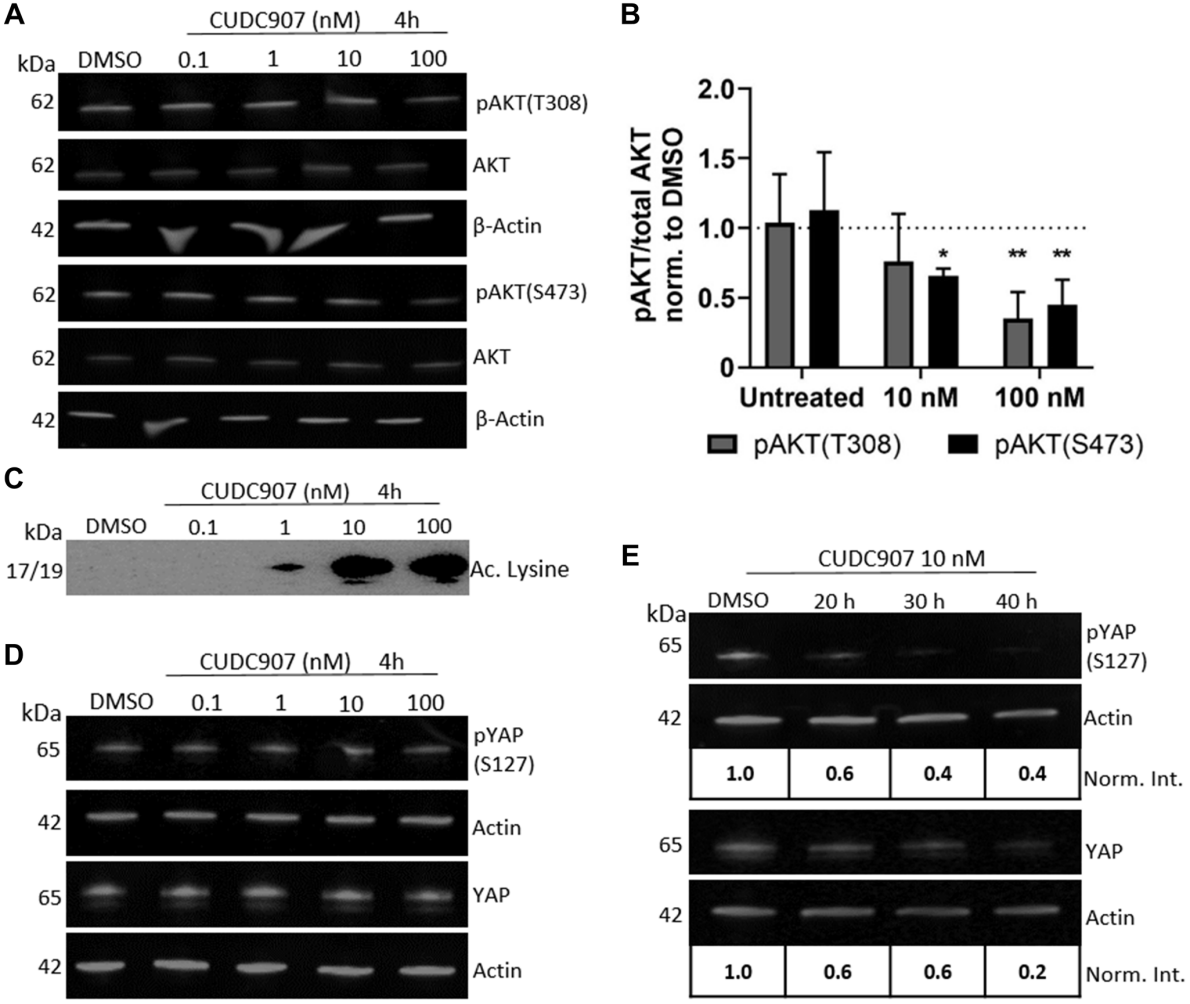
CUDC907 inhibits PI3K and HDAC activity in human MD-SC. (**A**) Representative Western blot and (**B**) quantification of pAKT levels in HS01 cells grown with CUDC907 as indicated for 4 h. ^*^ *p* < 0.05, ^**^ *p* < 0.01 compared to DMSO (dotted line), *n* = 3 independent experiments. (**C**) Representative Western blots of acetylated lysine in HS01 cells grown with CUDC907 as indicated for 4 h. (**D**) Phospho- and total YAP in HS01 cells grown as indicated, and (**E**) in the presence of 10 nM CUDC907 for indicated times. Norm. Int.- ratio of protein/actin, normalized to DMSO control. Differences in color in C due to chemiluminescent imaging (all other blots fluorescent).

CUDC907 promoted dose dependent decreases in pAKT at both T308 and S473 (Figure 3A and 3B) with no change in total AKT levels in human HS01 cells. Quantification of the ratio of pAKT to AKT revealed that a 4 h exposure to 10 nM CUDC907 decreased pAKT (T108 and S473) by more than 50% of controls (Figure 3B). A 4 h exposure of HS01 cells to 1 nM CUDC907 was sufficient to increase acetylated lysine levels (Figure 3C). Levels of phospho- and total FAK, and phospho- and total pERK1/2 were unchanged in HS01 cells treated with CUDC907 (Supplementary Figure 1). YAP has been identified as an important regulator of cell size and survival and its function is negatively regulated by merlin [13]. Temporal changes in phospho- and total YAP levels in HS01 cells treated with CUDC907 were also studied. Whereas CUDC907 did not reduce phospho- or total YAP at 4 h of exposure at any dose, 10 nM CUDC907 decreased both pYAP and total YAP by 40% of controls by 20 h. This effect increased through 40 h of treatment with a reduction of 60% compared to controls (Figure 3D and 3E).

### CUDC907 slowed implant growth compared to vehicle in a nerve allograft model

**Figure 4:**
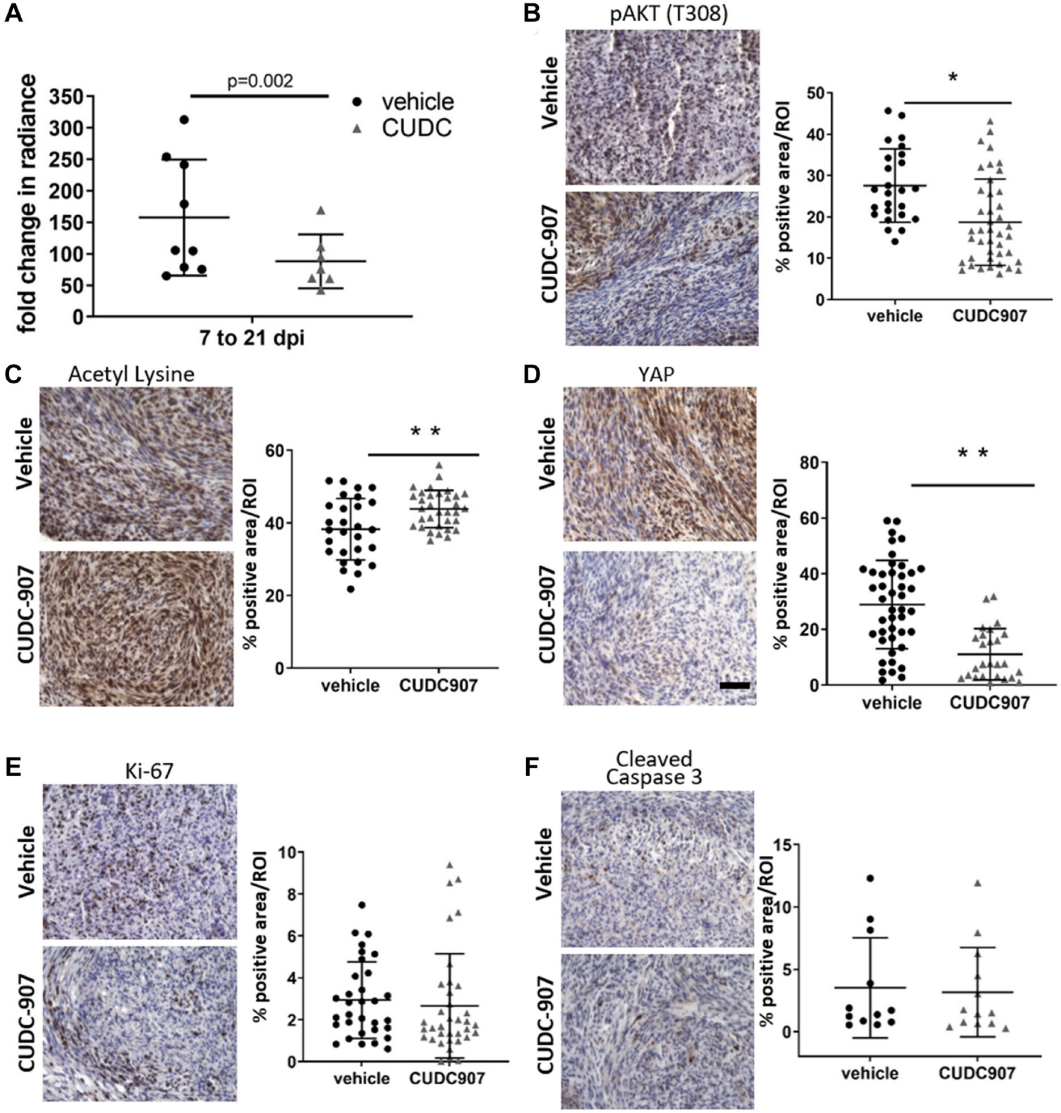
CUDC907 slows intraneural growth of mouse MD-SC. Vehicle (*n* = 9) and CUDC907 (*n* = 8) treated mice were used in a three week study. (**A**) Tumor radiance. (**B**–**F**) Representative images of immunohistochemical analysis of grafts with quantification. Images are 400× magnification; scale bar (in F): 50 μm. CUDC907 (B) significantly decreased pAKT, (C) increased acetyl lysine and (D) decreased total YAP in grafts, but did not significantly modulate expression of (E) Ki-67 or (F) cleaved caspase 3. Data points are individual fields from at least four fields/graft from 3–4 grafts with mean ± SD. Black bars indicate significant difference between groups. ^*^ *P* < 0.05, ^**^ *p* < 0.01, ^***^ *p* < 0.0001 compared to DMSO controls.

To assess *in vivo* efficacy of CUDC907, a validated sciatic nerve allograft model was used [24, 25]. Luciferase-expressing mouse MD-SCs (MS01-Luc) were injected into the right sciatic nerve of NSG mice. Successful grafting was confirmed by bioluminescent imaging 7 days post-injection (Supplementary Figure 2). Mice were assigned to vehicle (*n* = 9) and CUDC907 (*n* = 8, 25 mg/kg) treatment groups and dosed for 14 consecutive days. Bioluminescent imaging showed that grafts in CUDC907-treated mice grew significantly more slowly than those in vehicle-treated mice (Figure 4A). Grafts in the treated group had a 44% reduction in fold increase in average radiance over the 14-day treatment. Grafts displayed heterogeneity in tissue morphology (Supplementary Figure 3), and for protein markers assessed immunohistochemically. However, significantly lower pAKT levels (Figure 4B), significantly higher acetyl lysine expression (Figure 4C), and a dramatic decrease in total YAP levels (Figure 4D) were observed in CUDC907-treated grafts compared to controls. Significant differences between groups for Ki67 and cleaved caspase 3 staining intensities were not observed (Figure 4E and 4F).

### CUDC907 reduced cell viability and promoted apoptosis in primary VS cells

**Figure 5:**
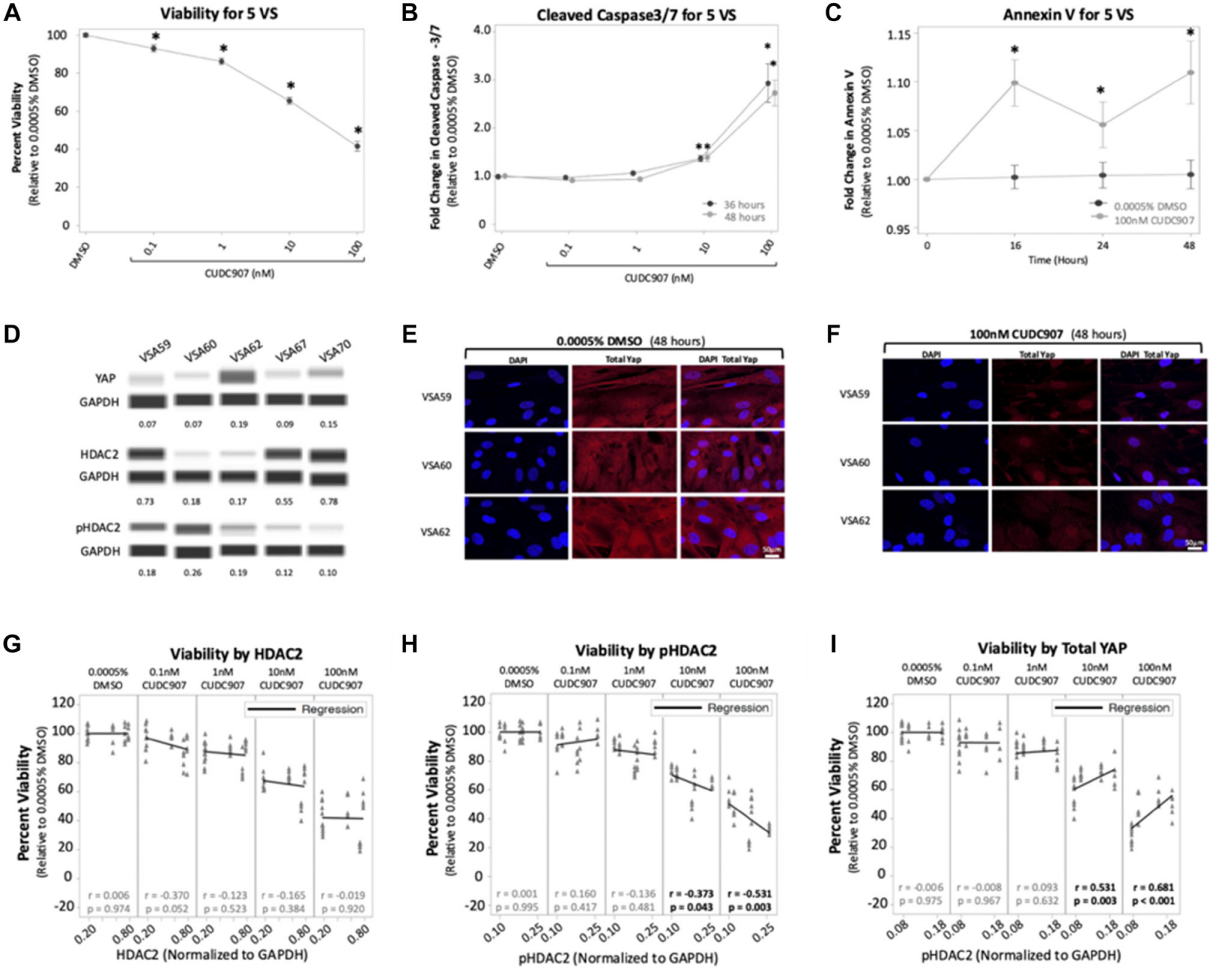
CUDC907 reduces viability and induces caspase cleavage in vestibular schwannomas. (**A**) CUDC907 causes a dose-dependent reduction in viability at 72 hrs. (**B**) CUDC907 at 10 and 100 nM promoted significant increases in cleaved caspase-3/7 at 36 and 48 hrs compared to 0.0005% DMSO. (**C**) CUDC907 at 100 nM induces more Annexin V expression at 16 h compared to 0.0005% DMSO. (**D**) Naïve tumor chunks from five VS show variable expression of YAP, HDAC2, and pHDAC2. Protein levels were normalized to GAPDH. (**E**–**F**) Representative confocal images from three VS reveal 100 nM CUDC907 significantly reduced cytoplasmic YAP at 48 hours compared to 0.0005% DMSO. (**G**–**I**) Regression analysis for normalized protein levels and viability. There was no correlation between HDAC2 and viability, but there was a moderate, inverse relationship between pHDAC2 and viability when VS cells were treated with 10 and 100 nM of CUDC907. At those concentrations, there was also a moderate, positive relationship between total YAP and viability. Data shown as mean ± SEM. ^*^ *p* < 0.05.

To examine the effect of CUDC907 on human VS cells, primary cultures established from five VS were treated with 0.0005% DMSO and CUDC907 (0.1–100 nM) and assays for viability, cleaved caspase-3/7, and annexin V were performed. All five tumors demonstrated NF2 mutations on whole exome sequencing, of which one (VSA62) was from a patient with a germline NF2 variant (Supplementary Table 2, [26]). Overall, CUDC907 caused a dose-dependent reduction in viability with 100 nM CUDC907 causing a 56% reduction (*p* < 0.05) at 72 h across 5 VS (Figure 5A). Individually, cells from the five VS responded remarkably to CUDC907 with all tumors achieving >20% reduction in viability compared to DMSO when treated with only 10 nM CUDC907 (Supplementary Figure 4A).

Treatment with 100 nM CUDC907 increased cleaved caspase-3/7 activity in VS cells analyzed as a group at 36 and 48 h, by approximately 2.1- and 2.9-fold, respectively, compared to vehicle (Figure 5B). Assessed individually, cells from the five VS demonstrated significant increases in cleaved caspase-3/7 to 100 nM of CUDC907, with mean fold increases of 1.3–6.8 and 1.8–5.5, respectively (Supplementary Figure 4B). Although individual VS showed increases in mean annexin V expression at 16 hrs (Supplementary Figure 4C), the increases were not significant for each VS. However, pooled data from the five tumors showed that CUDC907 (100 nM) induced a 8% increase in annexin V over DMSO controls at 16 h that was statistically significant (Figure 5C).

### CUDC907 reduced total YAP and increased nuclear p21 levels in VS cells

To determine whether CUDC907 modulated YAP levels, immunoblotting was performed with tissue lysates from the five VS. VS expressed varying amounts of YAP (Figure 5D, top). YAP immunocytochemistry revealed that VS cells from all five VS expressed YAP in the cytoplasm and nucleus (Supplementary Figure 5). Cytoplasmic staining was greatly reduced by exposure of VS cells to 100 nM CUDC907 for 24 h. Both cytoplasmic and nuclear YAP staining drastically decreased by 48 h of exposure to 100 nM CUCD907 (Figure 5E and 5F, Supplementary Figure 5). CUDC907 (100 nM CUDC907 for 24 h) also induced nuclear p21 expression in cells from all five VS, consistent with a G1 cell cycle arrest (Supplementary Figure 6).

### Viability response to CUDC907 correlated with pHDAC2 and total YAP levels in VS cells

To examine the relationship between HDAC expression and viability, immunoblotting for HDAC2 and pHDAC2 were conducted on VS tissue and normalized to GAPDH. VS expressed varying amounts of HDAC2 and pHDAC2 protein (Figure 5D, middle and bottom rows). There was no significant correlation between HDAC2 expression in VS and viability responses *in vitro* when VS cells were treated with CUDC907 (0.1–100 nM; Figure 5G). However, there was a significant moderate, inverse correlation between pHDAC2 expression in VS tissue and viability responses when VS cells were exposed to 10 nM (*r* = −0.37, *p* = 0.04) and 100 nM (*r* = −0.53, *p* = 0.003) CUDC907 (Figure 5H) but not to lower concentrations. Viability response to CUDC907 did not correlate with the pHDAC2/HDAC ratio (data not shown).

There was also a significant moderate, positive correlation between total YAP expression in VS tissue and viability responses when VS cells were exposed to 10 nM (*r* = 0.53, *p* = 0.003) and 100 nM (*r* = 0.68, *p* < 0.001) CUDC907 (Figure 5I).

## DISCUSSION

We screened a library of nearly 200 PI3K pathway inhibitors using a robust schema that included two isogenic pairs of human normal and schwannoma model cells and multiple cell viability and death assays. We prioritized drugs with selectivity for human MD-SCs over their isogenic normal parental cells. CUDC907 had amongst the lowest IG_50_ (low nM) and the highest selectivity window (5–100 fold). Only four inhibitors induced caspase-3/7 cleavage in human schwannoma model cells at low nanomolar doses; CUDC907 was chosen for further investigation.

The results of phenotypic and molecular studies obtained using human schwannoma model cells, VS primary cells, and an orthotopic allograft model are in good agreement, providing strong evidence that CUDC907 is a remarkable drug candidate for NF2. Together, they indicate that CUDC907 causes a G1 cell cycle arrest and triggers rapid pro-apoptotic signaling in human schwannoma model cells as well as VS cells. Live imaging studies showed that 1 nM and 10 nM CUDC907 promoted caspase-3/7 cleavage. Cleaved caspase-7, but not caspase-3, was observed in Western blots of CUDC-treated human MD-SCs. Moreover, flow cytometry assays confirmed increased populations of apoptotic and dead cell populations, and a G1 cell cycle arrest in human MD-SCs following a 24 h exposure to 100 nM CUDC907.

As expected for a dual HDAC/PI3K inhibitor, CUDC907 reduced pAKT and increased acetylated lysine in human schwannoma model cells. This activity is not unexpected as suppressed YAP expression and resultant loss of cancer cell viability has been reported previously for HDAC inhibitors in other grafted tumor models [27]. This effect of CUDC907 on YAP expression is of particular importance, because merlin is an upstream negative regulator of YAP and loss of merlin is associated with increased YAP expression and contributes to schwannomagenesis [28–30].

In addition, our findings using human MD-SCs were corroborated in cultured primary VS cells. In the five unique VS tested, exposure to CUDC907 produced a decrease in viability of nearly 56% of control. CUCD907 also induced p21 nuclear accumulation and cell death through caspase-3/7 cleavage. The effects of CUDC907 on viability and caspase cleavage across VS cells are robust. We have not observed this consistent response of VS cells to other candidate compounds nor the induction of caspase cleavage in any of our previous studies [31].

Total YAP, HDAC2, and pHDAC2 were assessed as potential biomarkers for response to CUDC907 in VS cells. Two relationships were identified: (1) a moderate, inverse relationship between pHDAC2 expression and viability, and (2) a moderate, positive relationship between total YAP expression and viability. These findings suggest that CUDC907 may be more effective at reducing viability of VS cells that express higher levels of pHDAC2, as expected from its HDAC inhibitory properties. The relationship between higher total YAP and higher cell viability following CUDC907 treatment was unexpected given the dramatic reduction in cytoplasmic and nuclear YAP on immunocytochemistry. However, this may be explained because VS cells may suppress YAP gene expression indirectly through HDAC inhibition, and VS cells may not exist in a state of active YAP addiction [27]. In several cancer cell lines with YAP located predominantly in the nucleus, loss of YAP dramatically suppressed viability of cells – a concept termed active YAP addiction. The growth inhibitory effects of YAP suppression were blunted in other cancer cell lines where YAP localizes predominantly in the cytoplasm, as seen in our five VS. Our sample size is small but provide evidence that pHDAC2 may be a potential biomarker to predict VS response to CUDC907 and further research is warranted to confirm this in other HDAC and pHDAC subtypes.

The orthotopic schwannoma model used here is a validated pre-clinical model of NF2 schwannomas. It has been used to assess efficacy of the VEGF inhibitor bevacizumab, the mTORC1 inhibitor rapamycin [32], and the ALK inhibitor crizotinib [33]. We observed a 44% reduction in graft size by radiance measurements within two weeks of daily CUDC907 (25 mg/kg) treatment compared to vehicle. Immunohistochemical analyses confirmed inhibition of PI3K and HDAC activity and reduced YAP staining in CUDC907-treated grafts compared to vehicle. We were unable to measure a statistically significant reduction in Ki67-positive cells or increased cleaved caspase-3 expression in CUDC907-treated grafts at the study’s end, though this may be due to the time point chosen and the pharmacokinetics of CUDC907. Additional studies will assess cell cycle progression and alternative cell death processes. However, the results of our cell-based studies suggest that cell death could have contributed to the decreased growth rate of mouse MD-SCs in CUDC907-treated mice.

CUDC907 was pre-clinically evaluated in several cell culture models as well as in two NF2 schwannoma and meningioma models by the Synodos for NF2 Consortium [34]. CUDC907 was among the top three effective compounds from the schwannoma cell-based screens contributed by us (using two cell lines studied here). Primary component analysis in the previous study determined that cell type and species of origin contributed more significantly to differences in treatment response than merlin status [34]. Analysis of merlin status as a single variable in this study confirms merlin-null specificity of CUDC907’s effect in several cell lines and species. In the Synodos study, CUDC907 reduced tumor size in a xenograft meningioma model by 55% compared to controls. It however failed to significantly slow schwannoma development in a genetically engineered mouse (GEM) NF2 model (Postn*Cre*;*Nf2*^fl/fl^) in which mice naturally develop schwannomas in dorsal root and cranial ganglia and have progressive hearing loss; although these are variable in time and rate of development [35]. GEM mice at six months of age received 25 mg/kg/day CUDC907 equal to the dose used here for twelve weeks. Possible explanations for the different schwannoma drug efficacy results are many and include differences in the animal strains used (immune competent vs. immune deficient), the mode and timing of tumor formation, treatment protocols, and sensitivity of the outcome measures for tumor growth. The GEM model used caliper measurements of the size of four dorsal root ganglions per animal at the end of treatment and thus could have missed early effects [34, 35].

Another HDAC inhibitor, AR42, has been studied in VS cells and a similar mouse schwannoma allograft model [36]. Our findings are consistent with those reported for AR-42 but differ in IC_50_ and the timing of appearance of apoptotic cells. The IC_50_ for CUDC907 is approximately 100-fold lower than AR42 in primary human VS and *Nf2-*deficient mouse schwannoma cells, which may be related to synergistic effects of CUDC907 on both PI3K and HDAC pathways. AR42 also promoted cell death after prolonged treatment (3–6 days) *in vitro* and 45 days in their mouse allograft study. With CUDC907, we noted apoptotic cells and cleaved caspase at 24 h in human schwannoma model and within 36–48 h in VS cells. Our inability to detect cleaved caspase in treated allografts may be time dependent or suggest non-canonical apoptotic processes and requires further investigation.

The mechanism by which CUDC907 induced cell cycle arrest and apoptosis in schwannoma model cells is likely associated with HDAC, PI3K/AKT, and YAP inhibition. Further investigation is necessary to fully understand the role of each pathway in the response to this inhibitor. Evidence for an early effect due to loss of HDAC activity is the following: (1) apoptotic human MD-SC were observed beginning at 16 h of treatment with 1 nM CUDC907 (Figure 1C), (2) loss of viability in VS cells correlated with higher levels of baseline pHDAC2 [20, 37–39], and lastly, (3) the kinome signature of the same line of human MD-SCs treated with CUDC907 more closely resembled the kinome of panobinostat-treated rather than omipalisib-treated HS01 cells [34]. Panobinostat is an HDAC inhibitor whereas omipalisib is a PI3K/mTOR inhibitor [40, 41]. We have not observed apoptosis of human schwannoma model cells treated with other Src or MEK inhibitors [42]. Although previous studies support HDAC inhibition as a mechanism for reduced YAP activity, additional molecular studies are needed to delineate the relationship between HDAC and PI3K inhibition, loss of YAP, and induction of apoptosis in human MD-SC [27, 31, 43]. Similarly, YAP phosphorylation, localization, and degradation are important aspects of YAP signaling and activity and will be assessed in future work; however, effects on total YAP expression were consistent in our models and have been correlated with disease progression and severity in several cancer types [44].

In summary, we demonstrated that CUDC907 reduced the activity of three major signaling pathways in NF2 schwannomas (HDAC, PI3K, and YAP) and consistently reduced viability and induced apoptosis in several schwannoma cell models and in all five genetically unique primary VS studied. These consistent results offer the possibility that CUDC907 will promote schwannoma regression in patients with diverse *NF2* mutations and support clinical evaluation of CUDC907 for NF2-associated schwannomas and potentially other cancers driven by *NF2* pathogenic variants [45]. Current use of this drug in clinical trials for other indications reveals clinical interest in multi-modal drugs over monotherapies.

## MATERIALS AND METHODS

### Cell culture

Primary human Schwann cells (SC), HS11 and HS13, were purchased from ScienCell (ScienCell, Cat#1700, Lots7228 and 21544, respectively). Merlin expression was suppressed in HS11 using lenti-*NF2*-shRNA (Sigma-Mission) to create HS01, as previously reported [46]. Merlin expression was knocked out in HS13 by CRISPR/Cas9 with *NF2*sg1 (Broad Institute) to create human merlin-deficient SCs (MD-SC), HS05. Human SCs were cultured on CellBIND dishes (Corning) in complete Schwann cell media (SCM) from ScienCell (basal medium plus 5% fetal bovine serum (FBS), growth supplements, and 1X-penicillin/streptomycin). Mouse MD-SCs (MS01) were generated and transduced with lenti-PGK V5-Luc-Neo as previously reported [46, 47]. MS01 cells were cultured on CellBIND dishes in N2 medium (F12, Dulbecco’s modified eagle medium [DMEM], and 1% N2 supplement). All cell lines were routinely tested for *Mycoplasma* (Lookout *Mycoplasma* PCR Detection Kit, Sigma).

### Human VS cultures and whole exome sequencing

Five patients with VS were consented for tumor banking through a University of Miami Institutional Review Board-approved protocol (#20150637). Vestibular schwannoma (VS) were harvested at the time of surgery and placed in chilled DMEM (Gibco). VS were divided into 1mm chunks and enzymatically dissociated with collagenase (150 U/ml) and dispase II (2.5 μg/ml) in DMEM overnight at 37°C and 5% CO_2_. Tissues were triturated and single cells were cultured on flasks coated with 0.01% poly-L-ornithine (PLO; Sigma) and laminin (25 μg/ml; ThermoFisher) in Schwann cell media (SCM) from ScienCell.

DNA was extracted from blood-derived monocytes and VS tissues using DNeasy Blood and Tissue Kit (Qiagen), per manufacturer’s instructions. Whole exome sequencing and analysis were performed at the Center for Genome Technology in the John P. Hussman Institute for Human Genomics at the University of Miami Miller School of Medicine. Briefly, 1 μg of genomic DNA was used as input for the SureSelectXT Human All Exon V7 capture library kit (Agilent) and sequencing performed on the Illumina NovaSeq 6000 in 100 basepair paired end reactions for ~100× average exome coverage. Raw sequencing FASTQs were processed through a bioinformatics pipeline consistent with the Genome Analysis Toolkit (GATK) best practices [48], including alignment to the GRCh38 human reference genome build using bwa-mem version 0.7.12, removal of duplicate reads with using Picard version 2.1.1, and base quality recalibration with GATK version 3.7. CalculateHsMetrics in Picard version 2.1.1 was used for quality control to ensure appropriate exome coverage was achieved. Germline variant calls were made with the GATK version 3.7 HaplotypeCaller. Somatic variants were determined from using the somatic function implemented in VarScan version 2.4.4 [49]. All variant calls were annotated using ANNOVAR, as previously described [50].

### Cell viability assay and drug library

Human SCs (1,000 cells/well) and mouse SCs (2,000 cells/well) were seeded in 384-well CellBIND plates (Corning) in phenol red-free growth medium. After 12 h, compounds from the PI3K/mTOR/AKT library (SelleckChem L2800) were diluted in dimethyl sulfoxide (DMSO) to 10 uM and added to three replicate wells for 48–72 h. CyQuant (ThermoFisher) and CellTiter-Fluor (Promega) were used according to manufacturer’s protocol to measure cell viability. IG_50_ indicates the inhibitor concentration required to reduce cell viability by 50% at 72 hours of treatment.

VS cells (5,000 cells/well) were seeded in 384-well CellBIND plates pre-coated with 0.01% poly-L-ornithine and laminin (25 μg/ml; ThermoFisher) in DMEM with 10% FBS. After 24 h, CUDC907 (diluted in DMSO) or 0.0005% DMSO in DMEM supplemented with 10% fetal bovine serum (FBS; Seradigm) and 1% penicillin-streptomycin (ThermoFisher) was added to 5–6 replicate wells for 72 h. Cell viability was measured with CellTiter-Glo (Promega) and GloMax^®^ Discover System (Promega) following manufacturer’s instructions.

### Membrane asymmetry assay

HS01 cells were seeded at 250,000 cells/well in 6-well CellBIND (Corning) plates, grown to ~80% confluency, and treated with CUDC907 for 24 h. Cells were harvested with 0.05% trypsin, washed, and resuspended in Hanks Balanced Salt Solution. The Violet Ratiometric Asymmetry Assay (Invitrogen) was used per the manufacturer’s instructions. Apoptotic, live and dead cell populations were measured by flow cytometry on Cytoflex (Beckman Coulter) and analyzed by CytExpert software (Beckman Coulter).

### Cell cycle analysis

HS01 cells were seeded at 250,000 cells/well in 6-well CellBIND (Corning) plates, grown to ~80% confluency, and treated with CUDC907 for 24 h. EdU (10 μM; Click-iT EdU kit; Molecular Probes, ThermoFisher) was added during the last 3 h. Cells were harvested with 0.05% trypsin, stained with violet live/dead stain (ThermoFisher), and permeabilized. DNA labeling with FxCycle stain (ThermoFisher) was conducted according to manufacturer’s protocol. Cell populations were identified by flow cytometry on CytoFlex (Beckman Coulter) and analyzed by CytExpert software (Beckman Coulter).

### Cell death assays

Human (1,000 cells/well) and mouse (2,000 cells/well) MD-SCs were seeded in phenol red-free growth medium in four replicate wells/condition of a 384-well CellBIND (Corning) plate. After ~13 h, drugs (diluted in DMSO) at three concentrations (the IG_50_ and lower and higher concentrations) and Incucyte^®^ Caspase-3/7 Green Apoptosis Assay Reagent (Sartorius) were added to wells. Wells were imaged using the Incucyte^®^ S3 Live-Cell Analysis system (Sartorius) for 72 h. Phase and green fluorescent images were collected every 4 h and analyzed using the integrated basic analyzer software.

Primary human VS cells were treated with CUDC907 as described for viability assays. The Caspase-Glo^®^ 3/7 Assay (Promega) was used to detect cleaved caspase-3/7 at 36 and 48 h, and the RealTime-Glo™ Annexin V Apoptosis Assay (Promega) was performed to detect phosphatidylserine over 48 h, following manufacturer’s protocols.

### Western blots/antibodies

HS01 cells were seeded at 250,000 cells/well in 6-well CellBIND (Corning) plates, grown to ~80% confluency, then treated with CUDC907 for indicated times. Cells were extracted with 4% sodium dodecyl sulfate (SDS), 0.01% bromophenol blue, 10% glycerol and 100 mmol/L dithiothreitol. Antibodies were used from Cell Signaling Technology: rabbit anti-cleaved caspase 7 (1:500; #8438); rabbit anti-cleaved caspase 3 (1:1000, #9664); rabbit anti-acetylated lysine (1:1000; #9814); mouse anti-actin (1:30,000; #3700); rabbit anti-pFAK(Y397) (1:1000; #8556), mouse anti-FAK (1:500; #3285); rabbit anti-pERK (1:1000; #4370); mouse anti-ERK (1:500; #9107); rabbit anti-pAKT (T308) (1:1000; #29655); mouse anti-AKT (1:1000; #29205); rabbit anti-pAKT (S473) (1:1000; #4060); rabbit anti-YAP (1:1000; #14074); rabbit anti-pYAP (S127) (1:1000; #4911). Primary antibodies were diluted in 1:1 tris-buffered saline-0.1% Tween and Odyssey Blocking Buffer or 5% milk and incubated overnight at 4°C. Secondary antibodies were diluted in same solution and incubated for 45 minutes at room temperature. Western blots were imaged on the LI-COR Odyssey Imaging System (LI-COR Biosciences) or ChemiDoc (Bio-Rad) and quantified on ImageJ (NIH).

### Capillary electrophoresis-based simple western assay

VS tissues were processed with radioimmunoprecipitation assay buffer (ThermoFisher; #89900), phosphatase inhibitors (ThermoFisher; #1862495) and protease inhibitors (Sigma; P8340) prior to sonication (Misonix) on ice. Protein was isolated and quantified using the bicinchoninic acid protein assay (Pierce). Chemiluminescence-based capillary electrophoresis Simple Western assays were performed per manufacturer’s protocol (Jess Simple Western; ProteinSimple). With the exception of YAP (2.4pg/capillary), 1.2pg of protein was loaded per capillary. Primary antibodies used were rabbit anti-YAP (1:50; Cell Signaling, #14074), mouse anti-HDAC2 (1:100; Invitrogen, MA5-18061), rabbit anti-pHDAC2(S394) (1:100; Abcam, ab75602), and rabbit anti-glyceraldehyde 3-phosphate dehydrogenase housekeeping protein (GAPDH; 1:150; Cell Signaling, #2118). Anti-rabbit and anti-mouse secondary horseradish peroxidase (HRP) antibodies (ready-to-use reagent; Bio-techne) were used. Protein expression was quantified using Compass for SW (version 6.0.06) and normalized to glyceraldehyde 3-phosphate dehydrogenase (GAPDH).

### Immunohistochemistry for VS

Primary VS cells were seeded at 10,000 cells/well in SCM on 16-well culture slides precoated with 0.01% PLO and laminin at 5% CO_2_ and 37°C. After 24 h, cells were treated with CUDC907 (100 nM) or 0.0005% DMSO (vehicle) in D10 media. Cells were fixed, permeabilized and blocked for 2 h at RT. Slides were exposed to primary antibodies overnight at 4°C, secondary antibodies at RT for 2 h, and DAPI nuclear stain (4′,6-diamidino-2-phenylindole; ab104139, Abcam) for 15 minutes. Slides were cover-slipped with anti-fade mounting medium (Sigma). Primary antibodies used were rabbit anti-YAP (1:200; Cell Signaling, #14074) and rabbit anti-p21 (1:100; ThermoFisher, MA5-14949) and donkey anti-rabbit IgG conjugated to AlexaFluor 594 (1:200; ThermoFisher). Images were obtained with the Leica SP5 Inverted Confocal Microscope (40X oil immersion lens) and assessed qualitatively.

### Nerve allograft

Male and female *NOD.Cg-Prkdcscid Il2rgtm1Wjl/SzJ* (NSG) mice were bred in house and all care and use was approved by the University of Central Florida (UCF) Institutional Animal Care and Usage Committee (IACUC; #20-165). The UCF animal facility is accredited by the Association for Assessment and Accreditation of Laboratory Animal Care. A total of 17 mice (7 and 8 weeks old) were implanted with MS01-*Luc* as previously described [24, 46]. Mice were imaged with an *In Vivo* Imaging System (IVIS, Perkin-Elmer) and peak radiance measurements (typically 8–15 minutes post-injection) were used to assign mice to treatment groups ensuring even distribution of initial tumor burden and sex between groups. Mice were dosed daily by oral gavage starting on day 8. Mice (*n* = 9) received vehicle (40% Kollisolv PEG400, 16% (2-Hydroxypropyl)-β-cyclodextrin) while mice (*n* = 8) received 25 mg/kg CUDC-907 solubilized in vehicle. Mice were imaged every 7 days and were euthanized after 14 days of treatment. All mice completed the study with consistent weight and no indication of illness. Necropsies were performed; no organ or tissue damage was observed. Although tumor segments were successfully dissected for histological processing, we were unable to reliably extract whole tumors for analysis of weight or volume due to changes in nerve tissue consistency after allograft tumor formation.

### Histology and immunohistochemistry

Grafts were placed in 4% paraformaldehyde immediately after dissection and fixed overnight at 4°C followed by standard paraffin processing. Samples were embedded and 5 μm sagittal sections were collected. Sections were stained with hematoxylin and eosin (H&E). Slides were deparaffinized and rehydrated. Antigen retrieval reaction was performed by heating sections in Antigen Unmasking Solution (pH 6.0, Vector Labs) in a 100°C water bath for 20 minutes. Endogenous peroxide activity was blocked with BLOXALL solution (Vector Labs) at room temperature for 10 minutes. After blocking for 1 h with 5% normal goat serum in PBS, primary antibodies were applied and slides were incubated overnight at 4°C. Primary antibodies used were rabbit anti-T308pAKT (1:70; Abcam, ab38449), rabbit anti-acetyl lysine (1:100; Abcam, ab80178), rabbit anti-Ki67 (1:200; Abcam, ab16667), and rabbit anti-cleaved caspase-3 (1:500; Cell Signaling, #9664). After primary staining with indicated antibodies, sections were washed and the ImmPRESS HRP Anti-Rabbit IgG Polymer Detection Kit (Vector Labs) was used followed by ImmPACT DAB HRP substrate (Vector Labs) to develop colorimetric signal. Sections were counterstained with hematoxylin and mounted with Permount (Fisher). Sections were imaged with a Keyence BZ-X800. Several regions of interest (ROI) were identified throughout the sections and images were collected at 400× magnification. Staining was quantified using the IHC toolbox plugin for ImageJ (NIH, v1.53e). At least two sections per sample were used for each protein target, with at least four ROIs imaged per section to account for tissue heterogeneity.

### Statistical analysis

GraphPad Prism Version 7.04 and SAS Version 9.4 were used for statistical analysis. Paired *t*-tests were used to compare western blot quantifications between CUDC907 and DMSO conditions and IVIS radiance fold change between CUDC907- and vehicle-treated groups. Two-way analysis of variance (ANOVA) with Bonferroni correction was used to compare treatment groups for cell cycle analysis and violet ratiometric membrane asymmetry assays. A Mann-Whitney test was used for immunohistochemical quantification analysis. Western blots, cell cycle analysis, violet ratiometric analysis and IncuCyte caspase-3/7 cleavage assays were repeated three times. For viability, cleaved caspase-3/7, and annexin assays with primary VS cells, 95% confidence intervals were calculated to determine differences between the five tumors and conditions. Linear regression analyses with Pearson’s correlation coefficient were performed to identify trends in viability response, as related to baseline HDAC2 and pHDAC2 expression in VS tumors. Statistical significance was set at *p* < 0.05.

## SUPPLEMENTARY MATERIALS



## Abbreviations

AKT: protein kinase B
ALK: anaplastic lymphoma kinase
DMEM: Dulbecco’s modified eagle medium
DMSO: dimethyl sulfoxide
EGFR: epidermal growth factor receptor
ERK: extracellular signal-regulated kinase
FAK: focal adhesion kinase
FBS: fetal bovine serum
GAPDH: glyceraldehyde 3-phosphate dehydrogenase
GATK: genome analysis tool kit
GEM: genetically engineered mouse
HDAC: histone deacetylase
HRP: horseradish peroxidase
IVIS: *in vivo* imaging system
LATS: large tumor suppressor kinase
LOPAC: library of pharmacologically active compounds
MAPK: mitogen-activated protein kinase
MD-SC: merlin deficient Schwann cell
MEK: mitogen-activated protein kinase kinase
mTOR: mammalian target of rapamycin
NF2: neurofibromatosis type 2
PI3K: phosphoinositide-3 kinase
PLO: poly-L-ornithine
ROI: region of interest
SC: Schwann cell
SCM: complete Schwann cell media
VEGF: vascular endothelial growth factor
VS: vestibular schwannoma
WT-SC: wildtype Schwann cell
YAP: yes-associated protein

## References

[1] Blakeley JO , Plotkin SR . Therapeutic advances for the tumors associated with neurofibromatosis type 1, type 2, and schwannomatosis. Neuro Oncol. 2016; 18:624–38. 10.1093/neuonc/nov200. 26851632 PMC4827037

[2] Dinh CT , Nisenbaum E , Chyou D , Misztal C , Yan D , Mittal R , Young J , Tekin M , Telischi F , Fernandez-Valle C , Liu XZ . Genomics, Epigenetics, and Hearing Loss in Neurofibromatosis Type 2. Otol Neurotol. 2020; 41:e529–37. 10.1097/MAO.0000000000002613. 32150022 PMC7547625

[3] Li J , Wang Q , Zhang M , Zhang G , Zhang S , Hui X . Malignant Transformation in Vestibular Schwannoma: Clinical Study With Survival Analysis. Front Oncol. 2021; 11:655260. 10.3389/fonc.2021.655260. 33937063 PMC8079768

[4] Evans DG , Birch JM , Ramsden RT , Sharif S , Baser ME . Malignant transformation and new primary tumours after therapeutic radiation for benign disease: substantial risks in certain tumour prone syndromes. J Med Genet. 2006; 43:289–94. 10.1136/jmg.2005.036319. 16155191 PMC2563223

[5] Cosetti MK , Golfinos JG , Roland JT Jr . Quality of Life (QoL) Assessment in Patients with Neurofibromatosis Type 2 (NF2). Otolaryngol Head Neck Surg. 2015; 153:599–605. 10.1177/0194599815573002. 25779467

[6] Aboukais R , Zairi F , Bonne NX , Baroncini M , Schapira S , Vincent C , Lejeune JP . Causes of mortality in neurofibromatosis type 2. Br J Neurosurg. 2015; 29:37–40. 10.3109/02688697.2014.952266. 25152998

[7] Baser ME , Friedman JM , Aeschliman D , Joe H , Wallace AJ , Ramsden RT , Evans DG . Predictors of the risk of mortality in neurofibromatosis 2. Am J Hum Genet. 2002; 71:715–23. 10.1086/342716. 12235555 PMC378530

[8] Lu VM , Ravindran K , Graffeo CS , Perry A , Van Gompel JJ , Daniels DJ , Link MJ . Efficacy and safety of bevacizumab for vestibular schwannoma in neurofibromatosis type 2: a systematic review and meta-analysis of treatment outcomes. J Neurooncol. 2019; 144:239–48. 10.1007/s11060-019-03234-8. 31254266

[9] Karajannis MA , Legault G , Hagiwara M , Ballas MS , Brown K , Nusbaum AO , Hochman T , Goldberg JD , Koch KM , Golfinos JG , Roland JT , Allen JC . Phase II trial of lapatinib in adult and pediatric patients with neurofibromatosis type 2 and progressive vestibular schwannomas. Neuro Oncol. 2012; 14:1163–70. 10.1093/neuonc/nos146. 22844108 PMC3424212

[10] Karajannis MA , Legault G , Hagiwara M , Giancotti FG , Filatov A , Derman A , Hochman T , Goldberg JD , Vega E , Wisoff JH , Golfinos JG , Merkelson A , Roland JT , Allen JC . Phase II study of everolimus in children and adults with neurofibromatosis type 2 and progressive vestibular schwannomas. Neuro Oncol. 2014; 16:292–97. 10.1093/neuonc/not150. 24311643 PMC3895376

[11] Trofatter JA , MacCollin MM , Rutter JL , Murrell JR , Duyao MP , Parry DM , Eldridge R , Kley N , Menon AG , Pulaski K . A novel moesin-, ezrin-, radixin-like gene is a candidate for the neurofibromatosis 2 tumor suppressor. Cell. 1993; 72:791–800. 10.1016/0092-8674(93)90406-g. 8453669

[12] Rouleau GA , Merel P , Lutchman M , Sanson M , Zucman J , Marineau C , Hoang-Xuan K , Demczuk S , Desmaze C , Plougastel B . Alteration in a new gene encoding a putative membrane-organizing protein causes neuro-fibromatosis type 2. Nature. 1993; 363:515–21. 10.1038/363515a0. 8379998

[13] Morrison H , Sperka T , Manent J , Giovannini M , Ponta H , Herrlich P . Merlin/neurofibromatosis type 2 suppresses growth by inhibiting the activation of Ras and Rac. Cancer Res. 2007; 67:520–27. 10.1158/0008-5472.CAN-06-1608. 17234759

[14] Petrilli AM , Fernández-Valle C . Role of Merlin/NF2 inactivation in tumor biology. Oncogene. 2016; 35:537–48. 10.1038/onc.2015.125. 25893302 PMC4615258

[15] Zhang N , Bai H , David KK , Dong J , Zheng Y , Cai J , Giovannini M , Liu P , Anders RA , Pan D . The Merlin/NF2 tumor suppressor functions through the YAP oncoprotein to regulate tissue homeostasis in mammals. Dev Cell. 2010; 19:27–38. 10.1016/j.devcel.2010.06.015. 20643348 PMC2925178

[16] Zhou L , Hanemann CO . Merlin, a multi-suppressor from cell membrane to the nucleus. FEBS Lett. 2012; 586:1403–8. 10.1016/j.febslet.2012.03.016. 22595235

[17] Rong R , Tang X , Gutmann DH , Ye K . Neurofibromatosis 2 (NF2) tumor suppressor merlin inhibits phosphatidylinositol 3-kinase through binding to PIKE-L. Proc Natl Acad Sci U S A. 2004; 101:18200–5. 10.1073/pnas.0405971102. 15598747 PMC535703

[18] Jacob A , Lee TX , Neff BA , Miller S , Welling B , Chang LS . Phosphatidylinositol 3-kinase/AKT pathway activation in human vestibular schwannoma. Otol Neurotol. 2008; 29:58–68. 10.1097/mao.0b013e31816021f7. 18199958

[19] Petrilli AM , Fuse MA , Donnan MS , Bott M , Sparrow NA , Tondera D , Huffziger J , Frenzel C , Malany CS , Echeverri CJ , Smith L , Fernández-Valle C . A chemical biology approach identified PI3K as a potential therapeutic target for neurofibromatosis type 2. Am J Transl Res. 2014; 6:471–93. 25360213 PMC4212923

[20] Qian C , Lai CJ , Bao R , Wang DG , Wang J , Xu GX , Atoyan R , Qu H , Yin L , Samson M , Zifcak B , Ma AW , DellaRocca S , et al. Cancer network disruption by a single molecule inhibitor targeting both histone deacetylase activity and phosphatidylinositol 3-kinase signaling. Clin Cancer Res. 2012; 18:4104–13. 10.1158/1078-0432.CCR-12-0055. 22693356

[21] Ranganna K , Selvam C , Shivachar A , Yousefipour Z . Histone Deacetylase Inhibitors as Multitarget-Directed Epi-Drugs in Blocking PI3K Oncogenic Signaling: A Polypharmacology Approach. Int J Mol Sci. 2020; 21:8198. 10.3390/ijms21218198. 33147762 PMC7662987

[22] Rodrigues DA , Pinheiro PSM , Fraga CAM . Multitarget Inhibition of Histone Deacetylase (HDAC) and Phosphatidylinositol-3-kinase (PI3K): Current and Future Prospects. ChemMedChem. 2021; 16:448–57. 10.1002/cmdc.202000643. 33049098

[23] Chilamakuri R , Agarwal S . Dual Targeting of PI3K and HDAC by CUDC-907 Inhibits Pediatric Neuroblastoma Growth. Cancers (Basel). 2022; 14:1067. 10.3390/cancers14041067. 35205815 PMC8870466

[24] Turk AN , Byer SJ , Zinn KR , Carroll SL . Orthotopic xenografting of human luciferase-tagged malignant peripheral nerve sheath tumor cells for *in vivo* testing of candidate therapeutic agents. J Vis Exp. 2011; 2558. 10.3791/2558. 21460792 PMC3197311

[25] Giovannini M , Bonne NX , Vitte J , Chareyre F , Tanaka K , Adams R , Fisher LM , Valeyrie-Allanore L , Wolkenstein P , Goutagny S , Kalamarides M . mTORC1 inhibition delays growth of neurofibromatosis type 2 schwannoma. Neuro Oncol. 2014; 16:493–504. 10.1093/neuonc/not242. 24414536 PMC3956353

[26] Håvik AL , Bruland O , Myrseth E , Miletic H , Aarhus M , Knappskog PM , Lund-Johansen M . Genetic landscape of sporadic vestibular schwannoma. J Neurosurg. 2018; 128:911–22. 10.3171/2016.10.JNS161384. 28409725

[27] Han H , Yang B , Nakaoka HJ , Yang J , Zhao Y , Le Nguyen K , Bishara AT , Mandalia TK , Wang W . Hippo signaling dysfunction induces cancer cell addiction to YAP. Oncogene. 2018; 37:6414–24. 10.1038/s41388-018-0419-5. 30068939 PMC6294669

[28] Guerrant W , Kota S , Troutman S , Mandati V , Fallahi M , Stemmer-Rachamimov A , Kissil JL . YAP Mediates Tumorigenesis in Neurofibromatosis Type 2 by Promoting Cell Survival and Proliferation through a COX-2-EGFR Signaling Axis. Cancer Res. 2016; 76:3507–19. 10.1158/0008-5472.CAN-15-1144. 27216189 PMC4911274

[29] Mindos T , Dun XP , North K , Doddrell RD , Schulz A , Edwards P , Russell J , Gray B , Roberts SL , Shivane A , Mortimer G , Pirie M , Zhang N , et al. Merlin controls the repair capacity of Schwann cells after injury by regulating Hippo/YAP activity. J Cell Biol. 2017; 216:495–510. 10.1083/jcb.201606052. 28137778 PMC5294779

[30] White SM , Avantaggiati ML , Nemazanyy I , Di Poto C , Yang Y , Pende M , Gibney GT , Ressom HW , Field J , Atkins MB , Yi C . YAP/TAZ Inhibition Induces Metabolic and Signaling Rewiring Resulting in Targetable Vulnerabilities in NF2-Deficient Tumor Cells. Dev Cell. 2019; 49:425–43.e9. 10.1016/j.devcel.2019.04.014. 31063758 PMC6524954

[31] Fuse MA , Dinh CT , Vitte J , Kirkpatrick J , Mindos T , Plati SK , Young JI , Huang J , Carlstedt A , Franco MC , Brnjos K , Nagamoto J , Petrilli AM , et al. Preclinical assessment of MEK1/2 inhibitors for neurofibromatosis type 2-associated schwannomas reveals differences in efficacy and drug resistance development. Neuro Oncol. 2019; 21:486–97. 10.1093/neuonc/noz002. 30615146 PMC6422635

[32] Wong HK , Lahdenranta J , Kamoun WS , Chan AW , McClatchey AI , Plotkin SR , Jain RK , di Tomaso E . Anti-vascular endothelial growth factor therapies as a novel therapeutic approach to treating neurofibromatosis-related tumors. Cancer Res. 2010; 70:3483–93. 10.1158/0008-5472.CAN-09-3107. 20406973 PMC4785015

[33] Troutman S , Moleirinho S , Kota S , Nettles K , Fallahi M , Johnson GL , Kissil JL . Crizotinib inhibits NF2-associated schwannoma through inhibition of focal adhesion kinase 1. Oncotarget. 2016; 7:54515–25. 10.18632/oncotarget.10248. 27363027 PMC5342359

[34] Allaway R , Angus SP , Beauchamp RL , Blakeley JO , Bott M , Burns SS , Carlstedt A , Chang LS , Chen X , Clapp DW , Desouza PA , Erdin S , Fernandez-Valle C , et al, and Synodos for NF2 Consortium. Traditional and systems biology based drug discovery for the rare tumor syndrome neurofibromatosis type 2. PLoS One. 2018; 13:e0197350. 10.1371/journal.pone.0197350. 29897904 PMC5999111

[35] Gehlhausen JR , Park SJ , Hickox AE , Shew M , Staser K , Rhodes SD , Menon K , Lajiness JD , Mwanthi M , Yang X , Yuan J , Territo P , Hutchins G , et al. A murine model of neurofibromatosis type 2 that accurately phenocopies human schwannoma formation. Hum Mol Genet. 2015; 24:1–8. 10.1093/hmg/ddu414. 25113746 PMC4262489

[36] Bush ML , Oblinger J , Brendel V , Santarelli G , Huang J , Akhmametyeva EM , Burns SS , Wheeler J , Davis J , Yates CW , Chaudhury AR , Kulp S , Chen CS , et al. AR42, a novel histone deacetylase inhibitor, as a potential therapy for vestibular schwannomas and meningiomas. Neuro Oncol. 2011; 13:983–99. 10.1093/neuonc/nor072. 21778190 PMC3158011

[37] Tu T , Huang J , Lin M , Gao Z , Wu X , Zhang W , Zhou G , Wang W , Liu W . CUDC-907 reverses pathological phenotype of keloid fibroblasts *in vitro* and *in vivo* via dual inhibition of PI3K/Akt/mTOR signaling and HDAC2. Int J Mol Med. 2019; 44:1789–800. 10.3892/ijmm.2019.4348. 31545402 PMC6777681

[38] Sun K , Atoyan R , Borek MA , Dellarocca S , Samson ME , Ma AW , Xu GX , Patterson T , Tuck DP , Viner JL , Fattaey A , Wang J . Dual HDAC and PI3K Inhibitor CUDC-907 Downregulates MYC and Suppresses Growth of MYC-dependent Cancers. Mol Cancer Ther. 2017; 16:285–99. 10.1158/1535-7163.MCT-16-0390. 27980108

[39] Kotian S , Zhang L , Boufraqech M , Gaskins K , Gara SK , Quezado M , Nilubol N , Kebebew E . Dual Inhibition of HDAC and Tyrosine Kinase Signaling Pathways with CUDC-907 Inhibits Thyroid Cancer Growth and Metastases. Clin Cancer Res. 2017; 23:5044–54. 10.1158/1078-0432.CCR-17-1043. 28600475 PMC6959516

[40] Singh A , Patel VK , Jain DK , Patel P , Rajak H . Panobinostat as Pan-deacetylase Inhibitor for the Treatment of Pancreatic Cancer: Recent Progress and Future Prospects. Oncol Ther. 2016; 4:73–89. 10.1007/s40487-016-0023-1. 28261641 PMC5315073

[41] Munster P , Aggarwal R , Hong D , Schellens JH , van der Noll R , Specht J , Witteveen PO , Werner TL , Dees EC , Bergsland E , Agarwal N , Kleha JF , Durante M , et al. First-in-Human Phase I Study of GSK2126458, an Oral Pan-Class I Phosphatidylinositol-3-Kinase Inhibitor, in Patients with Advanced Solid Tumor Malignancies. Clin Cancer Res. 2016; 22:1932–39. 10.1158/1078-0432.CCR-15-1665. 26603258

[42] Petrilli AM , Garcia J , Bott M , Klingeman Plati S , Dinh CT , Bracho OR , Yan D , Zou B , Mittal R , Telischi FF , Liu XZ , Chang LS , Welling DB , et al. Ponatinib promotes a G1 cell-cycle arrest of merlin/NF2-deficient human schwann cells. Oncotarget. 2017; 8:31666–81. 10.18632/oncotarget.15912. 28427224 PMC5458238

[43] Zhang D , Damoiseaux R , Babayan L , Rivera-Meza EK , Yang Y , Bergsneider M , Wang MB , Yong WH , Kelly K , Heaney AP . Targeting Corticotroph HDAC and PI3-Kinase in Cushing Disease. J Clin Endocrinol Metab. 2021; 106:e232–46. 10.1210/clinem/dgaa699. 33000123 PMC8921634

[44] Zanconato F , Cordenonsi M , Piccolo S . YAP/TAZ at the Roots of Cancer. Cancer Cell. 2016; 29:783–803. 10.1016/j.ccell.2016.05.005. 27300434 PMC6186419

[45] Younes A , Berdeja JG , Patel MR , Flinn I , Gerecitano JF , Neelapu SS , Kelly KR , Copeland AR , Akins A , Clancy MS , Gong L , Wang J , Ma A , et al. Safety, tolerability, and preliminary activity of CUDC-907, a first-in-class, oral, dual inhibitor of HDAC and PI3K, in patients with relapsed or refractory lymphoma or multiple myeloma: an open-label, dose-escalation, phase 1 trial. Lancet Oncol. 2016; 17:622–31. 10.1016/S1470-2045(15)00584-7. 27049457 PMC5494693

[46] Thaxton C , Bott M , Walker B , Sparrow NA , Lambert S , Fernandez-Valle C . Schwannomin/merlin promotes Schwann cell elongation and influences myelin segment length. Mol Cell Neurosci. 2011; 47:1–9. 10.1016/j.mcn.2010.12.006. 21182951 PMC3129596

[47] Fuse MA , Plati SK , Burns SS , Dinh CT , Bracho O , Yan D , Mittal R , Shen R , Soulakova JN , Copik AJ , Liu XZ , Telischi FF , Chang LS , et al. Combination Therapy with c-Met and Src Inhibitors Induces Caspase-Dependent Apoptosis of Merlin-Deficient Schwann Cells and Suppresses Growth of Schwannoma Cells. Mol Cancer Ther. 2017; 16:2387–98. 10.1158/1535-7163.MCT-17-0417. 28775147

[48] Van der Auwera GA , Carneiro MO , Hartl C , Poplin R , Del Angel G , Levy-Moonshine A , Jordan T , Shakir K , Roazen D , Thibault J , Banks E , Garimella KV , Altshuler D , et al. From FastQ data to high confidence variant calls: the Genome Analysis Toolkit best practices pipeline. Curr Protoc Bioinformatics. 2013; 43:11.10.1–11.10.33. 10.1002/0471250953.bi1110s43. 25431634 PMC4243306

[49] Koboldt DC , Zhang Q , Larson DE , Shen D , McLellan MD , Lin L , Miller CA , Mardis ER , Ding L , Wilson RK . VarScan 2: somatic mutation and copy number alteration discovery in cancer by exome sequencing. Genome Res. 2012; 22:568–76. 10.1101/gr.129684.111. 22300766 PMC3290792

[50] Rong AJ , Gallo RA , Zhang MG , Doddapaneni R , Griswold AJ , Lee JY , Kurtenbach S , Dubovy SR , Tse DT , Pelaez D . Establishment and Characterization of a Novel Human Ocular Adnexal Sebaceous Carcinoma Cell Line. Transl Vis Sci Technol. 2021; 10:34. 10.1167/tvst.10.6.34. 34043754 PMC8161695

