# Supplementary material for: CUDC907, a dual phosphoinositide-3 kinase/histone deacetylase inhibitor, promotes apoptosis of NF2 Schwannoma cells
Source: Oncotarget. 2022 Jul 19;13:890–904. doi: 10.18632/oncotarget.28254 (PMC9295707; doi:10.18632/oncotarget.28254)
Supplement: Supplementary file 1 [file oncotarget-13-28254-s001.pdf]

CUDC907, a dual phosphoinositide-3 kinase/histone deacetylase inhibitor, promotes apoptosis of NF2 Schwannoma cells

SUPPLEMENTARY MATERIALS

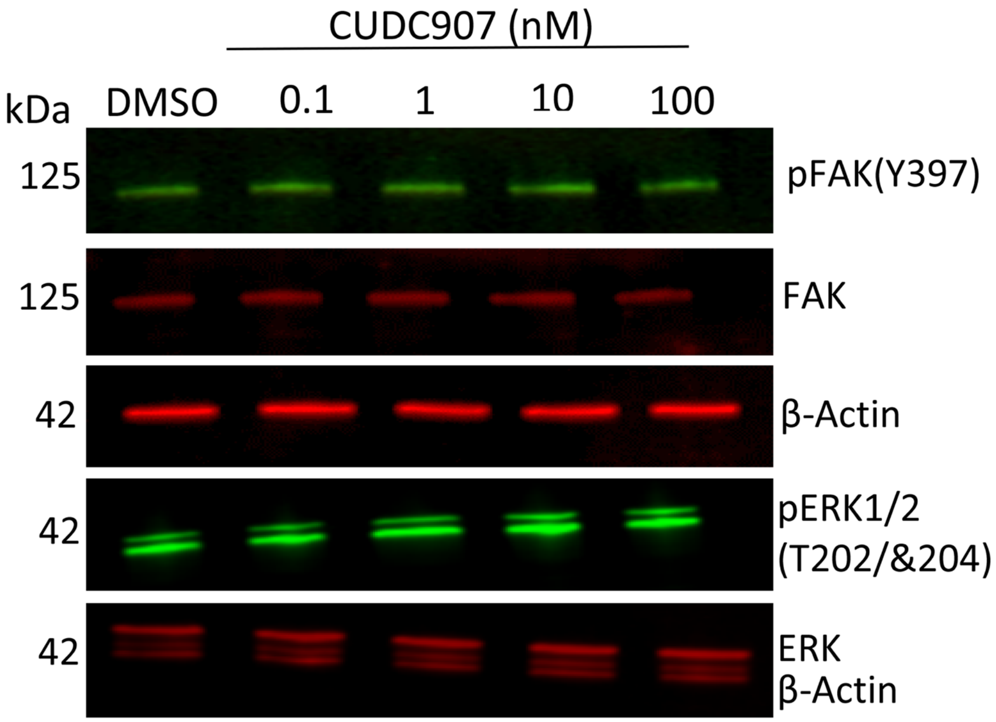

Supplementary Figure 1: Western blot analysis of CUDC907 treated HS01 cells (4 h) shows no inhibition of FAK or ERK.

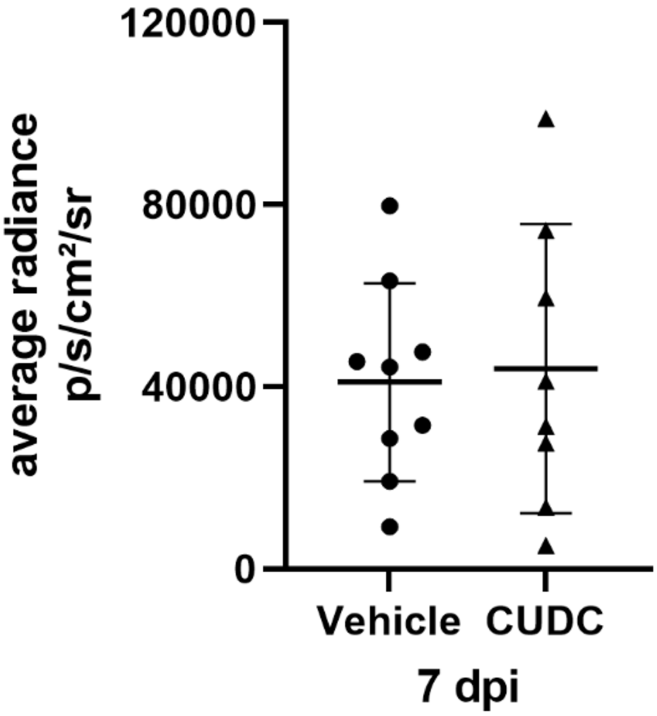

Supplementary Figure 2: Relative bioluminescent signals shown at 7 days post injection to indicate group similarity at treatment initiation (*p* = 0.82).

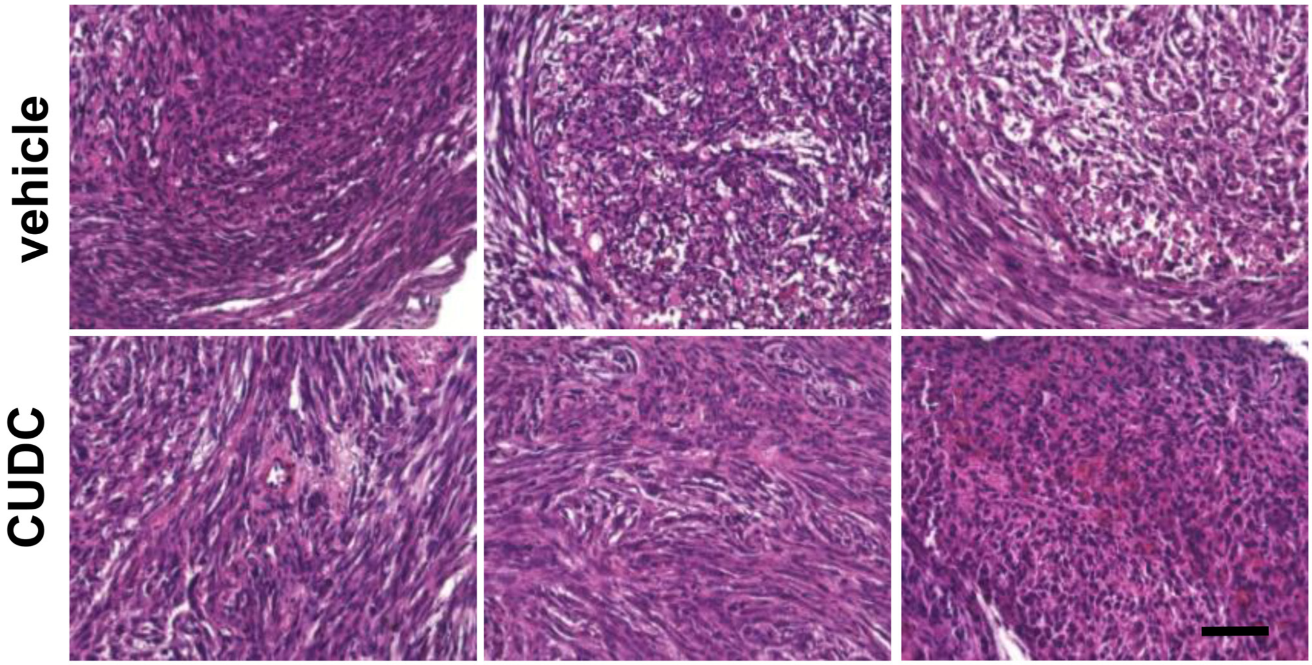

Supplementary Figure 3: Hematoxylin and eosin staining of grafts from sciatic allograft mouse model. Histological analysis was completed on five grafts from each condition. Representative images of H&E staining of each tumor at 400× magnification. Scale bar (bottom right): 50 µm.

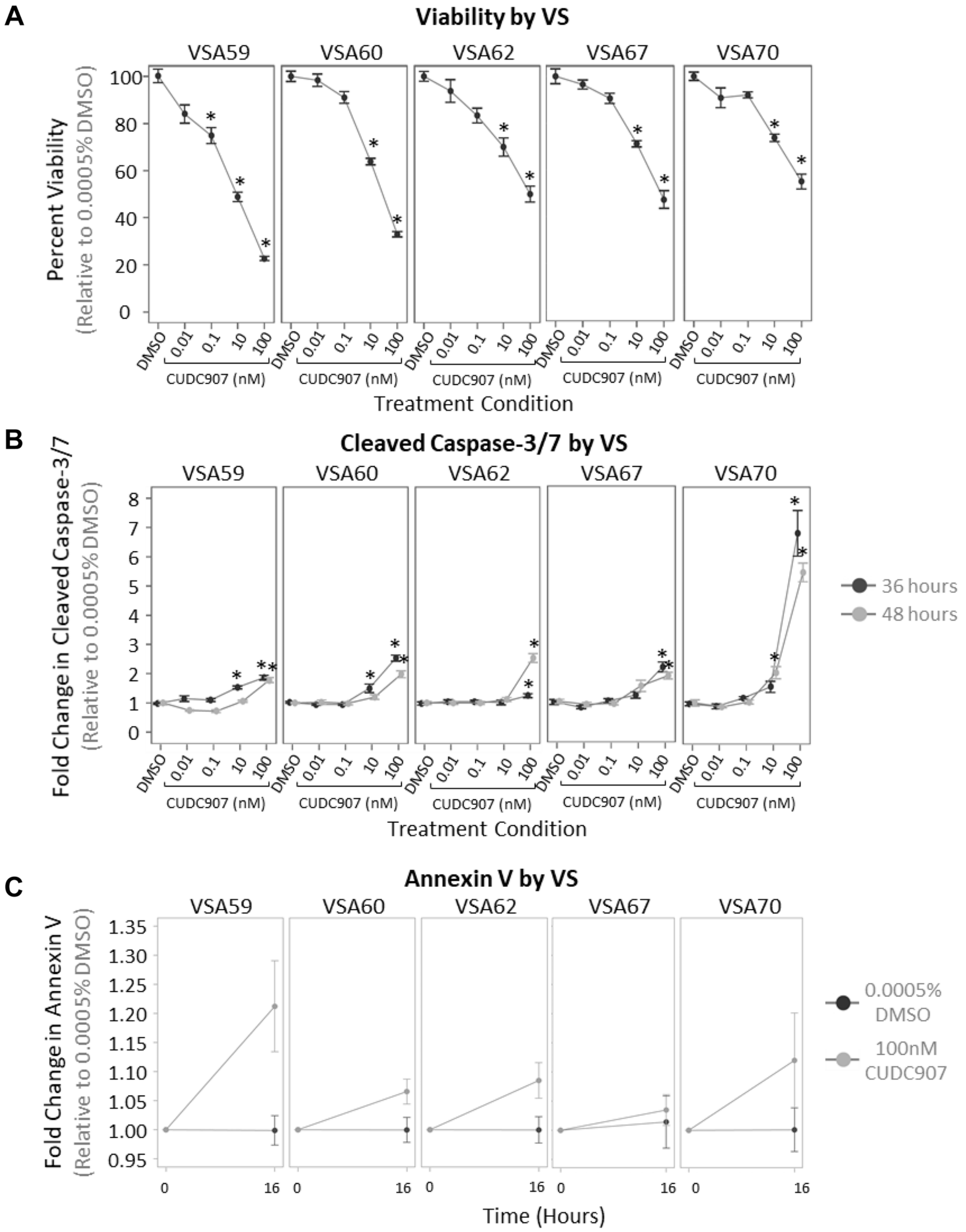

Supplementary Figure 4: Viability, cleaved caspase-3/7, and annexin V in vestibular Schwannoma (VS) cells. (**A**) Percent Viability by VS. CUDC907 causes a dose-dependent reduction in viability in all five VS at 10 and 100 nM CUDC907. (**B**) Cleaved Caspase-3/7 Activity by VS. At 100 nM concentrations, CUDC907 initiated significant increases in cleaved caspase-3/7, when compared to 0.0005% DMSO in all five VS at 36 and 48 hours. (**C**) Annexin V Expression by VS. All five VS demonstrated increases in mean annexin V at 16 hours compared to 0.0005% DMSO; however, they did not reach significance within individual VS. Bar = mean. Error bar = standard error mean. $^{*}p < 0.05$.

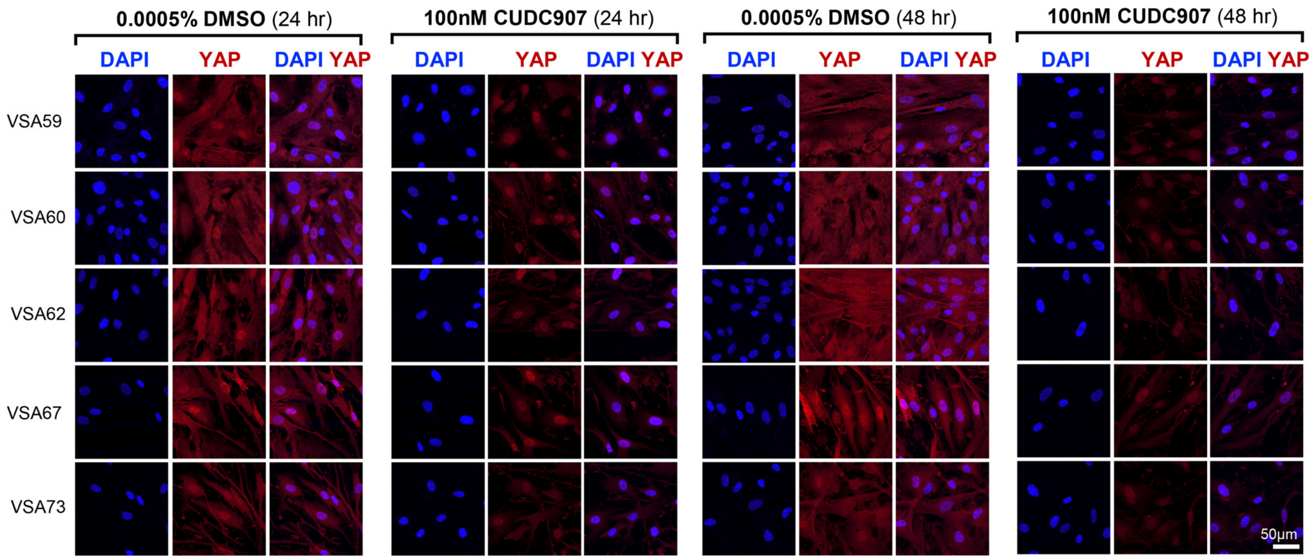

Supplementary Figure 5: Immunohistochemistry for YAP. CUDC907 (100 nM) reduced cytoplasmic expression of YAP in primary cells from five vestibular schwannomas, when compared to treatment with 0.0005% DMSO (vehicle) at both 24 and 48 hrs. CUDC907 also reduced nuclear YAP staining from 24 to 48 hours. DAPI represents nuclear staining.

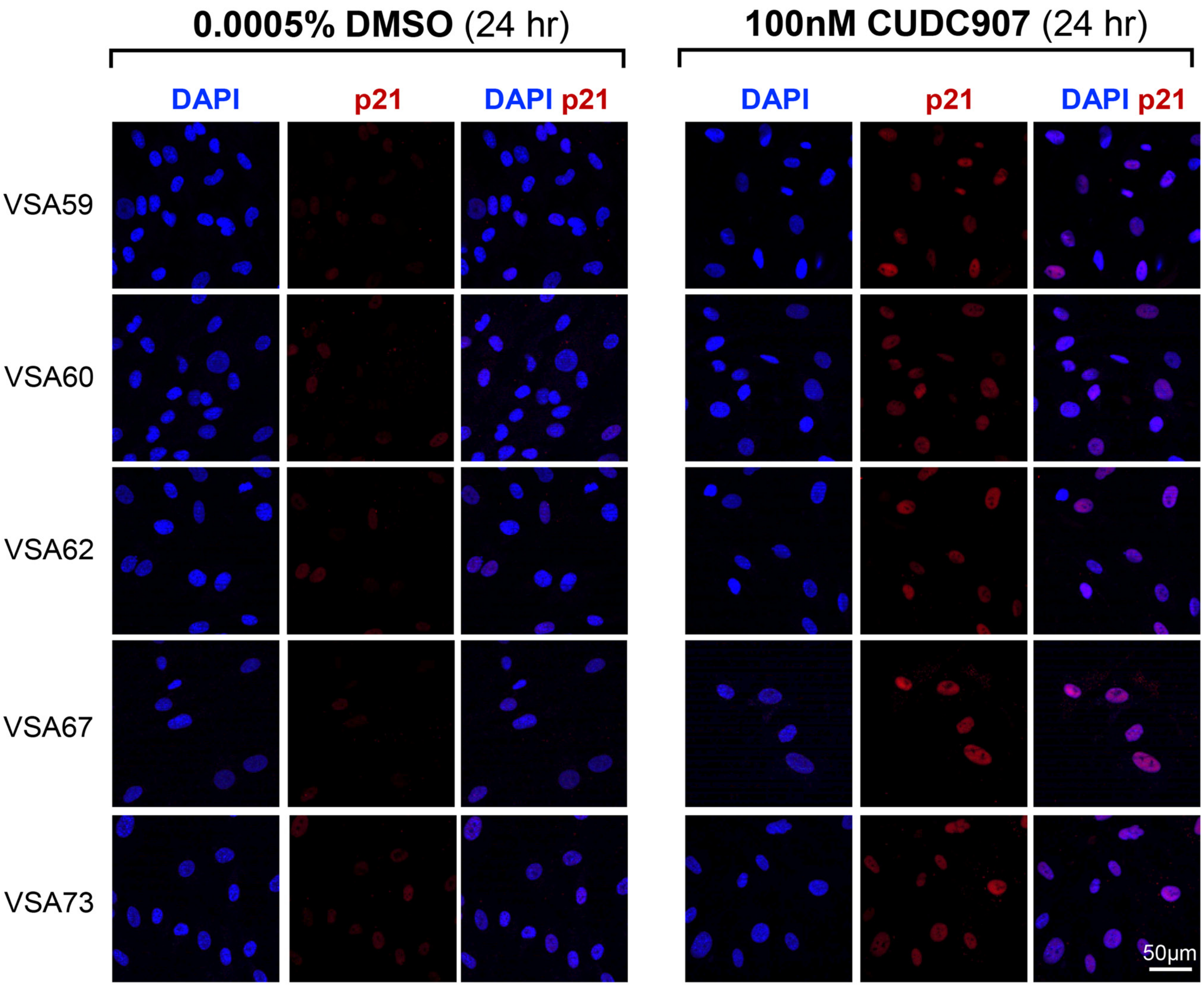

Supplementary Figure 6: Immunohistochemistry for p21. CUDC907 (100 nM) increased nuclear expression of p21 in primary cells from five vestibular schwannomas, when compared to treatment with 0.0005% DMSO (vehicle) at 24 hrs. DAPI represents nuclear staining.

Supplementary Table 1A: PI3K/mTOR/AKT pathway inhibitor library - 174 compounds (Selleckchem)

Compound ID	Target	Compound ID	Target	Compound ID	Target
Picfeltarraenin IA	AChE/PI3K/EGFR	TWS119	GSK-3β	A66	PI3Kα
Honokiol	Akt	Indirubin	GSK-3β	CH5132799	PI3Kα
TIC10 Analogue	Akt	TDZD-8	GSK-3β	Alpelisib (BYL719)	PI3Kα
Perifosine (KRX-0401)	Akt	IM-12	GSK-3β	HS-173	PI3Kα
Miransertib (ARQ 092) HCl	Akt	Tideglusib	GSK-3β	Serabelisib (INK-1117,MLN-1117,TAK-117)	PI3Kα
SC79	Akt	AR-A014418	GSK-3β	GDC-0326	PI3Kα
TIC10	Akt	1-Azakenpaullone	GSK-3β	PIK-93	PI3Kα, PI4KIIIβ
Triciribine	Akt, DNA synthesis inhibitor	CP21R7 (CP21)	GSK-3β	Buparlisib (BKM120, NVP-BKM120)	PI3Kα/β/δ/γ
Deguelin	Akt, PI3K	**CUDC-907**	**HDAC, PI3K**	Apitolisib (GDC-0980, RG7422)	PI3Kα/β/δ/γ, mTOR
AT13148	Akt, S6K, ROCK, PKA	Lupeol	Immunology & Inflammation	PI-103	PI3Kα/β/δ/γ, mTOR, DNA-PK
PHT-427	Akt,PDK1	Ridaforolimus (MK-8669, Deforolimus)	mTOR	SF2523	PI3Kα/γ, DNA-PK, BRD4, mTOR
**SC66**	**Akt/mTOR**	Rapamycin (Sirolimus)	mTOR	Pictilisib (GDC-0941)	PI3Kα/δ
A-674563	Akt1,CDK,PKA	Temsirolimus (CCI-779, NSC 683864)	mTOR	LY294002	PI3Kα/δ/β
Akti-1/2	Akt1/2	Everolimus (RAD001)	mTOR	Pilaralisib (XL147)	PI3Kα/δ/γ
MK-2206 2HCl	Akt1/2/3	WYE-354	mTOR	Taselisib (GDC 0032)	PI3Kα/δ/γ
Afuresertib (GSK2110183)	Akt1/2/3	AZD8055	mTOR	XL147 analogue	PI3Kα/δ/γ
Ipatasertib (GDC-0068)	Akt1/2/3	Torkinib (PP242)	mTOR	TGX-221	PI3Kβ
AZD5363	Akt1/2/3	WYE-125132 (WYE-132)	mTOR	AZD6482	PI3Kβ
AT7867	Akt1/2/3, p70S6K/PKA	WAY-600	mTOR	PI-3065	PI3Kβ

Supplementary Table 1B: PI3K/mTOR/AKT pathway inhibitor library - 174 compounds (Selleckchem)

Compound ID	Target	Compound ID	Target	Compound ID	Target
CCT128930	Akt2	Zotarolimus(ABT-578)	mTOR	GSK2636771	PI3Kβ
Uprosertib (GSK2141795)	Akt3	Sapanisertib (INK 128, MLN0128)	mTOR	AZD8186	PI3Kβ/δ
GSK690693	Aktt1/2/3	XL388	mTOR	AS-252424	PI3Kγ
Adenosine 5'-MP monohydrate	AMPK	Vistusertib (AZD2014)	mTOR	AS-604850	PI3Kγ
Euphorbiasteroid	AMPK	GDC-0349	mTOR	CAY10505	PI3Kγ
Dorsomorphin (Compound C) 2HCl	AMPK	CZ415	mTOR	IPI-549	PI3Kγ
Dorsomorphin (Compound C)	AMPK	CC-223	mTOR	3-Methyladenine (3-MA)	PI3Kγ, Vps34
KU-55933	ATM/ATR	Torin 2	mTOR, ATM/ ATR/ DNA-PK	TG100-115	PI3Kγ/δ
KU-60019	ATM/ATR	GSK1059615	mTOR, PI3Kα/β/δ/γ	TG100713	PI3Kγ/δ/α/β
VE-822	ATM/ATR	VS-5584 (SB2343)	mTOR, PI3Kα/β/δ/γ	PIK-293	PI3Kδ
AZ20	ATM/ATR	Gedatolisib (PF-05212384, PKI-587)	mTOR, PI3Kα/γ	Idelalisib (CAL-101, GS-1101)	PI3Kδ
AZD6738	ATM/ATR	Voxtalisib (XL765, SAR245409) Analogue	mTOR, PI3Kγ, DNA-PK	PIK-294	PI3Kδ
**CGK 733**	**ATM/ATR**	Omipalisib (GSK2126458)	mTOR,PI3K	IPI-3063	PI3Kδ
Mirin	ATM/ATR	BGT226 (NVP-BGT226)	mTOR,PI3K	Acalisib (GS-9820)	PI3Kδ
BAY 1895344 (BAY-1895344)	ATM/ATR	LY3023414	mTOR,PI3K, DNA-PK	CZC24832	PI3Kδ
AZD1390	ATM/ATR	Voxtalisib (XL765, SAR245409)	mTOR,PI3Kγ	Seletalisib (UCB-5857)	PI3Kδ
VE-821	ATM/ATR	OSI-027	mTORC1 & C2	AMG319	PI3Kδ
ETP-46464	ATM/ATR, mTOR	KU-0063794	mTORC1 &2	umbralisib (TGR-1202)	PI3Kδ
Schisandrin B (Sch B)	ATM/ATR,P-gp	Palomid 529 (P529)	mTORC1/2	GSK2292767	PI3Kδ

Supplementary Table 1C: PI3K/mTOR/AKT pathway inhibitor library - 174 compounds (Selleckchem)

Compound ID	Target	Compound ID	Target	Compound ID	Target
YU238259	DNA-PK	Torin 1	mTORC1/2	Nemiralisib (GSK2269557)	PI3Kδ
LTURM34	DNA-PK	HTH-01-015	NUAK, AMPK	ZSTK474	PI3Kδ
NU7026	DNA-PK	WZ4003	NUAK, AMPK	Tenalisib (RP6530)	PI3Kδ/γ
PP121	DNA-PK, mTOR, PDGFR,Src,VEGFR, Bcr-Abl	ATP	Others	H 89 2HCl	PKA, S6 Kinase
CC-115	DNA-PK,mTOR	Isorhamnetin 3-O-neohesperoside	Others	SF1670	PTEN
NU7441 (KU-57788)	DNA-PK,PI3K	Apigenin	P450 (CYP2C9)	bpV (HOpic)	PTEN
PIK-75 HCl	DNA-PK,PI3Kα	OSU-03012 (AR-12)	PDK-1	VO-Ohpic trihydrate	PTEN
Chrysophanic Acid	EGFR,mTOR	BX-795	PDK1,IκB/IKK	BI-D1870	S6 Kinase
5-Bromoindole	GSK-3	BX-912	PDK1,IκB/IKK	LY2584702 Tosylate	S6 Kinase
Bikinin	GSK-3	GSK2334470	PDK1,IκB/IKK	LY2584702	S6 Kinase
AZD2858	GSK-3	GNE-317	PI3K	LJH685	S6 Kinase
SB216763	GSK-3α/β	PF-4989216	PI3K	LJI308	S6 Kinase
CHIR-99021 (CT99021)	GSK-3α/β	AZD8835	PI3K	PF-4708671	S6K1 isoform
SB415286	GSK-3α/β	leniolisib(CDZ 173)	PI3K	Quercetin	Src,Sirtuin,PKC, PI3K
CHIR-99021 (CT99021) HCl	GSK-3α/β	Duvelisib (IPI-145, INK1197)	PI3K δ/γ	Notoginsenoside R1	TNF-α/ERK1/2, PI3K/Akt
CHIR-98014	GSK-3α/β	PF-04691502	PI3K(α/β/δ/γ), mTOR	**Piperlongumine**	**TrxR1, PI3K/Akt/ mTOR**
BIO-acetoxime	GSK-3α/β	GDC-0084	PI3K,mTOR	VPS34 inhibitor 1 (Cpd 19, PIK-III analogue)	VPS34, PI3K
LY2090314	GSK-3α/β	Miltefosine	PI3K/Akt	VPS34-IN1	Vps34, PI3K
AZD1080	GSK-3α/β	Bimiralisib (PQR309)	PI3K/mTOR	Autophinib	Vps34, PI3K
BIO	GSK-3α/β	YM201636	PI3Kfyve	PIK-III	VPS34, PI3Kδ

Supplementary Table 2: Whole exome sequencing (WES) for *NF2* gene mutations in vestibular schwannoma tumors

Sample	*NF2* Gene Mutations on Whole Exome Sequencing								
	Position (GRCh38)	Mutation Type	Blood DP	Blood VAF	Tumor DP	Tumor VAF	mRNA (NM_000268)	Protein (NP_000259)	Exon
VSA59	chr22:29636804	Frameshift deletion	106	0.00%	82	17.10%	c.168_177del	p.R57Gfs*63	2
	chr22:29673349	Frameshift deletion	93	0.00%	106	31.10%	c.1203delG	p.A402Pfs*24	12
VSA60	chr22:29642286	Splice Site mutation	60	0.00%	30	80.00%	c.447+1G>A	.	4
VSA62	*chr22:29661313	Stop-gain mutation	50	14.00%	47	12.80%	c.C784T	p.R262X	8
VSA67	chr22:29674892	Frameshift deletion	49	4.10%	23	39.10%	c.1397delG	p.R466Qfs	13
VSA70	chr22:29604112	Nonsynchronous single nucleotide variant	108	0.00%	72	18.10%	c.G114T	p.E38D	1

The five tumors demonstrated NF2 mutations on WES, consisting of frameshift deletions, splice site mutation, stop-gain mutation, and nonsynchronous single nucleotide variant. One patient with tumor VSA62 had a germline NF2 mutation causing a stop-gain mutation. Acronyms/Symbols: *germline mutation; Abbreviations: DP: read depth; GRCH38: Genome Reference Consortium Human Build 38; VAF: variant allele fraction.
